# Quantum Optical Effective-Medium Theory for Layered Metamaterials at Any Angle of Incidence

**DOI:** 10.3390/nano13020291

**Published:** 2023-01-10

**Authors:** Ehsan Amooghorban, Martijn Wubs

**Affiliations:** 1 Faculty of Science, Department of Physics, Shahrekord University, P.O. Box 115, Shahrekord 88186-34141, Iran; 2 Nanotechnology Research Group, Shahrekord University, P.O. Box 115, Shahrekord 88186-34141, Iran; 3 Department of Electrical and Photonics Engineering, Technical University of Denmark, 2800 Kgs. Lyngby, Denmark; 4 Center for Nanostructured Graphene, Technical University of Denmark, 2800 Kgs. Lyngby, Denmark; 5 NanoPhoton—Center for Nanophotonics, Technical University of Denmark, 2800 Kgs. Lyngby, Denmark

**Keywords:** loss-compensated metamaterials, effective-medium theory, quantum optics, 42.50.Ct, 42.50.Nn, 03.70.+k, 78.20.Ci, 78.67.Pt

## Abstract

The quantum optics of metamaterials starts with the question of whether the same effective-medium theories apply as in classical optics. In general, the answer is negative. For active plasmonics but also for some passive metamaterials, we show that an additional effective-medium parameter is indispensable besides the effective index, namely, the effective noise-photon distribution. Only with the extra parameter can one predict how well the quantumness of states of light is preserved in the metamaterial. The fact that the effective index alone is not always sufficient and that one additional effective parameter suffices in the quantum optics of metamaterials is both of fundamental and practical interest. Here, from a Lagrangian description of the quantum electrodynamics of media with both linear gain and loss, we compute the effective noise-photon distribution for quantum light propagation in arbitrary directions in layered metamaterials, thereby detailing and generalizing our previous work. The effective index with its direction and polarization dependence is the same as in classical effective-medium theories. As our main result, we derive both for passive and for active media how the value of the effective noise-photon distribution too depends on the polarization and propagation directions of the light. Interestingly, for *s*-polarized light incident on passive metamaterials, the noise-photon distribution reduces to a thermal distribution, but for *p*-polarized light it does not. We illustrate the robustness of our quantum optical effective-medium theory by accurate predictions both for power spectra and for balanced homodyne detection of output quantum states of the metamaterial.

## 1. Introduction

Metamaterials are known and studied for guiding and manipulating light in ways not seen in nature [1,2]. They consist of repeated designed subwavelength unit-cell structures that allow a description of the metamaterial in terms of effective optical parameters not found in natural materials, with negative-index metamaterials [1,3] as the prime example. In this Introduction, we discuss applications of metamaterials in quantum optics, and justify the need for a quantum optical effective-medium theory.

Applications in optics of metamaterials include flat superlenses [1,4,5,6,7] and sensors [8]. Metamaterials can constitute a material basis for applications of transformation optics [9] such as cloaking devices, which typically require graded-index media realized as graded-*effective*-index media.The properties of a metamaterial derive from an average of its constituting materials, which often involve both metals and dielectrics. There are different ways to determine the effective refractive index of a metamaterial, which is the topic of homogenization theory [10,11,12,13,14,15,16,17,18,19].

One important class of structures for which such averaging can produce truly new functionalities are the epsilon-near-zero (or ENZ) materials [20,21,22,23,24], in which light propagates with extremely small phases and long effective wavelengths, as has been realized also at visible wavelengths [25,26]. Dispersion-compensated metamaterials can also lead to new devices [27]. Loss-compensated metamaterials constitute another class of structures for which averaging over a unit cell can produce something truly new [4,28,29]: loss in one constituent can be compensated by linear gain in another, so as to produce metamaterials with lower or even vanishing effective loss. Partial loss compensation has been realized both in plasmonic waveguides [30,31] and in metamaterials [32]. Loss compensation is studied in the field of active plasmonics, tuneable metamaterials and Parity-Time (PT)-symmetric metamaterials with balanced amounts of gain and loss [33,34,35,36,37,38,39,40,41,42,43]. All mentioned applications of metamaterials are within the realm of classical electromagnetism.

Quantum plasmonics concerns the study of quantum optics with plasmons [44,45]. It is a stimulating question which of the mentioned applications of metamaterials can be transferred to quantum optics. Indeed, an increasing number of researchers is exploring how to manipulate quantum emitters and quantum states of light using metamaterials [46,47,48,49,50,51,52,53,54,55,56,57]. Vice versa, the exploration of how quantum states of light can be used to analyze metamaterial properties [58,59] belongs to the emerging field of quantum state spectroscopy [60].

The best known and important example of metamaterials with new functionality for quantum emitters are the hyperbolic metamaterials. Their effective epsilon is positive in one or two directions and negative-valued in the remaining direction(s) [46]. By taking the usual limit of infinitely small unit cells, the iso-frequency dispersion surfaces of such anisotropic bulk media become hyperbolic, with infinite associated local optical density of states. This nonphysical infinity indicates that the usual idealized description of metamaterials needs improvement for embedded quantum emitters, for example, by taking into account the nonlocality of the metallic response [61], or the finite size of either the unit cells [62] or the emitters [63]. Thus, quantum emitters embedded inside metamaterials provide a challenge for the effective-medium theories [64].

Quantum optics poses another lesser known challenge to metamaterials, even when probing metamaterials in the far field and when unit cells are much smaller than the operating wavelength: One can peform quantum optical experiments to tell apart two metamaterials even though they have the same shape and the same effective index [59]. In classical electrodynamics, this would be impossible, but in quantum optics this may even be possible with normally incident light on simple layered metamaterials [59]. This is because of quantum noise. Quantum mechanics poses a limit to the use of the common effective-index theories.

In principle, the ‘quantumness’ of light can survive the propagation through a metamaterial. In general, quantum states of light that propagate through absorbing or amplifying media will be affected by quantum noise associated with the loss [65,66,67,68,69] and gain [70,71,72]. This also applies to metamaterials. This does not mean that the concept of the effective index breaks down in quantum optics. On the contrary, in Ref. [59], we presented a quantum optical effective-index theory that accurately describes passive metamaterials and the more exotic metamaterials consisting of alternating layers both with gain. The theory also describes the quantum noise in these metamaterials, and can be seen as a direct extension of the usual effective-index theory.

However, for loss-compensated metamaterials, we found that the effective index sometimes underestimates the average quantum noise picked up in a unit cell, because loss can be compensated by gain but quantum noise due to loss cannot be compensated by quantum noise due to gain. Thus, effective descriptions of loss-compensated metamaterials based solely on the effective index break down in quantum optics. Nevertheless, an accurate quantum optical effective-medium theory of loss-compensated metamaterials is still possible, where, besides the usual effective, index an additional effective-medium parameter is introduced for loss-compensated metamaterials, namely, the effective noise photon distribution [59]. These results were obtained only for normally incident light on multilayer metamaterials.

Here, we generalize Ref. [59] in important ways by considering quantum optical effective-medium theories for *three-dimensional* light propagation in layered metamaterials. As is well-known in classical optics, *s*- and *p*-polarized light propagate qualitatively different in a layered medium. Analogously, we will here present surprisingly different effective noise-photon densities for *s*- and *p*-polarized light. Only for normal incidence will they coincide with each other and with the effective noise-photon density of the one-dimensional theory of Ref. [59]. We also address anew the question of whether it is only the loss-compensated metamaterials that require an additional effective-medium parameter.

During its life as a preprint [73], applications of the formalism and results as developed in an earlier version of this paper have already been applied in several works, including Refs. [74,75]. These concentrate on the realization of PT-symmetric optical systems and the propagation of quantum states of light through them.

The paper is organized as follows: In Section 2, we introduce the field quantization of media with both gain and loss, presenting what we believe is the shortest and simplest route from a Lagrangian to a phenomenological quantum electrodynamics based on the classical Green function. We use these results to derive, in Section 3, an input–output relation for planar multilayer dielectrics. In Section 4, we derive a quantum optical effective-index theory, while another effective theory, namely, a quantum optical effective-medium theory for both *s*- and *p*-polarized light, is introduced in Section 5. We discuss in Section 6 when these two theories will give the same predictions. We test power spectra predicted with both effective theories in Section 7, and, similarly, the predicted propagation of squeezed states of light through metamaterials in Section 8. We end with a discussion and conclusions in Section 9. Various technical details are presented in five appendices.

## 2. Field Quantization

With application to loss-compensated metamaterials in mind, here we derive a general expression for the quantized electric field after non-normal propagation through a bounded inhomogeneous dielectric medium that exhibits both loss and gain. Quantum-mechanical theories for electromagnetic wave propagation through lossy [65,66,67] or amplifying [70,71] dielectrics have been developed previously. We described media with both gain and loss in Ref. [76], where we used path-integral quantization techniques. Here, instead, we will not use path integrals and instead we give a simpler quantum electrodynamical description of media with both gain and loss, which is valid for arbitrary dielectric structures, including all non-magnetic metamaterials. The method has the advantage that there is a clear relation between the dielectric function of the dielectric medium and the more microscopic coupling parameters in the Lagrangian. This section results in a Macroscopic QED theory for arbitrary inhomogeneous media with both loss and gain. Its specific application to multilayer structures then follows in Section 3.

The quantum electrodynamics of a linearly lossy dielectric can be described by modeling the medium as a reservoir of three-dimensional harmonic oscillators that interacts with the electromagnetic field [65]. We also allow for the possibility that the medium is linearly amplifying in some finite regions of space, with gain ($Im\left[\varepsilon \left(\omega \right)\right]\equiv \varepsilon_{I}\left(\omega \right)<0$) in one or more finite-frequency windows. Linear gain can be modeled as the coupling of the electromagnetic field to a continuum of inverted harmonic oscillators [77,78].

We introduce our model for optical media with both gain and loss by first specifying its Lagrangian density in real space [76]

(1) $$L=L_{\mathrm{EM}}+L_{e}+L_{\mathrm{int}},$$

where the first term $L_{\mathrm{EM}}$ has the standard form $L_{\mathrm{EM}}=\frac{1}{2}\varepsilon_{0}E^{2}\left(x,t\right)-\frac{1}{2\mu_{0}}B^{2}\left(x,t\right),$ describing the free electromagnetic field. There is gauge freedom to write the electric field E=−∂A/∂t−∇ϕ and the magnetic field B=∇×A in terms of the scalar and vector potentials ϕ and A. For convenience, we choose the Coulomb gauge in which the divergence of the vector potential vanishes by definition. The second term $L_{e}$ in Equation (1) denotes the internal dynamics of the linear medium, which we describe in terms of the frequency continua of the harmonic vector field $X_{\omega }\left(x,t\right)$ as

(2) $$\begin{array}{c} L_{e}=\frac{1}{2}\int_{0}^{\infty }d\omega \;\left[{{\dot{X}}^{2}}_{\omega }\left(x,t\right)-\omega^{2}X_{\omega }^{2}\left(x,t\right)\right]\;\mathrm{sgn}\left[\varepsilon_{I}\left(x,\omega \right)\right].\;\;\;\;\;\;\; \end{array}$$

We define the polarization field of the medium as

(3) $$\begin{array}{c} P\left(x,t\right)=\int_{0}^{\infty }d\omega \;g\left(x,\omega \right)X_{\omega }\left(x,t\right), \end{array}$$

and assume a linear coupling of the electromagnetic field with this field,

(4) $$L_{\mathrm{int}}\left(A,P,\phi \right)=A\left(x,t\right)\cdot \dot{P}\left(x,t\right)+\phi \nabla \cdot P.$$

The g(x,ω) in Equation (3) is assumed to be a real-valued scalar coupling function of the electromagnetic field to the spatially inhomogeneous medium. At positions and for frequencies for which $\varepsilon_{I}\left(x,\omega \right)$ is positive-valued, the medium is lossy and $X_{\omega }\left(x,t\right)$ is an oscillator to which electromagnetic energy is lost, whereas if $\varepsilon_{I}\left(x,\omega \right)$ has a negative value, then the medium is amplifying the electromagnetic signal. The latter is modeled with oscillators that are called ‘inverted’ because of the overall minus sign $\mathrm{sgn}[\varepsilon_{I}\left(x,\omega \right)]=-1$ in the material Lagrangian density Equation (2). The time derivative of the scalar potential ($\dot{\phi }$) does not appear in the Lagrangian density (1). This implies, in the first place, that the conjugate momentum associated with the scalar potential ϕ is identically zero. Secondly, the scalar potential (by its Euler–Lagrange equation) can be expressed in terms of other degrees of freedom by Poisson’s equation $\varepsilon_{0}\nabla^{2}\phi =\nabla \cdot P$. The solution is $\phi \left(x,t\right)={\left(4\pi \varepsilon_{0}\right)}^{-1}\int dx^{\prime }\nabla^{\prime }\cdot P\left(x^{\prime },t\right)/\left|x-x^{\prime }\right|$. The scalar potential is thereby eliminated, and a reduced Lagrangian is obtained where only the vector potential **A**, the harmonic vector field $X_{\omega }$ and their time derivatives appear. To this end, the free electromagnetic field part and its interaction part are rewritten as

(5a) $$\begin{array}{ccc} L_{\mathrm{EM}}\left(A\right) & = & \frac{1}{2}\varepsilon_{0}{\dot{A}}^{2}\left(x,t\right)-\frac{1}{2\mu_{0}}{\left(\nabla \times A\left(x,t\right)\right)}^{2}, \end{array}$$

(5b) $$\begin{array}{ccc} L_{\mathrm{int}}\left(A,P\right) & = & A\left(x,t\right)\cdot \dot{P}\left(x,t\right)+\frac{1}{8\pi \varepsilon_{0}}\int dx^{\prime }\frac{\nabla \cdot P\left(x,t\right)\nabla^{\prime }\cdot P\left(x^{\prime },t\right)}{|x-x^{\prime }|}, \end{array}$$

while the material Lagrangian density (2) stays without any changes because there is no term including the scalar potential ϕ. Here, and in the following, we take the medium to be non-magnetic, and for extensions to magnetodielectrics we refer to Ref. [76]. The Lagrangian (1), with the vector potential A, and the continua of the polarization operator $X_{\omega }$ can be used as canonical fields with the following corresponding canonically conjugate fields

(6a) $$\begin{array}{ccc} -\varepsilon_{0}E\left(x,t\right) & \equiv & \frac{\delta L}{\delta \dot{A}\left(x,t\right)}=\varepsilon_{0}\dot{A}\left(x,t\right), \end{array}$$

(6b) $$\begin{array}{ccc} Q_{\omega }\left(x,t\right) & \equiv & \frac{\delta L}{\delta {\dot{X}}_{\omega }\left(x,t\right)}=g\left(\omega ,x\right)A\left(x,t\right)+\mathrm{sgn}\left[\varepsilon_{I}\left(x,\omega \right)\right]{\dot{X}}_{\omega }\left(x,t\right). \end{array}$$

Until now there is no difference with a classical description. We arrive at a quantum theory by taking the fields to be quantum fields (operator vector fields) that satisfy non-vanishing equal-time commutation relations with their canonically conjugate fields. Apart from the subtlety with the sign functions in Equation (6b), which discriminate between the frequency intervals where there is gain and loss, this canonical quantization of the fields can proceed in a standard fashion by demanding equal-time commutation relations

(7a) $$\begin{array}{ccc} \left[A_{i}\left(x,t\right),-\varepsilon_{0}E_{j}\left(x^{\prime },t\right)\right] & = & i\hbar \;\delta_{ij}\delta^{⊥}\left(x-x^{\prime }\right), \end{array}$$

(7b) $$\begin{array}{ccc} \left[X_{\omega ,i}\left(x,t\right),Q_{\omega^{\prime },j}\left(x^{\prime },t\right)\right] & = & i\hbar \;\;\delta_{ij}\;\delta \left(\omega -\omega^{\prime }\right)\;\delta^{3}\left(x-x^{\prime }\right),\;\;\;\;\;\;\;\;\;\;\; \end{array}$$

while all other equal-time commutators vanish. Using the Lagrangian (1) and the expressions for the canonical conjugate variables in Equation (6), we obtain the Hamiltonian density

(8) $$\begin{array}{ccc} H(x,t) & = & \frac{1}{2}\varepsilon_{0}E^{2}\left(x,t\right)+\frac{B^{2}\left(x,t\right)}{2\mu_{0}}\\ & & +\frac{1}{2}\int_{0}^{\infty }d\omega \;\mathrm{sgn}\left[\varepsilon_{I}\left(x,\omega \right)\right]\{{\left(Q_{\omega }\left(x,t\right)-g\left(\omega ,x\right)A\left(x,t\right)\right)}^{2}+\omega^{2}X_{\omega }^{2}\left(x,t\right)\}. \end{array}$$

Maxwell’s equations can now be obtained from the Heisenberg equations of motion for the vector potential and the transverse electric field and from the commutation relation Equation (7),

(9a) $$\begin{array}{ccc} \dot{A}\left(x,t\right) & = & -E(x,t), \end{array}$$

(9b) $$\begin{array}{ccc} \varepsilon_{0}\dot{E}\left(x,t\right) & = & \mu_{0}^{-1}\nabla \times \nabla \times A\left(x,t\right)-\dot{P}\left(x,t\right). \end{array}$$

Using the definitions $D=\varepsilon_{0}E+P$ for the displacement field and and $H=B/\mu_{0}$ for the magnetic field strength, Equation (9) results in $\dot{D}\left(x,t\right)=\nabla \times H\left(x,t\right)$ and $\dot{B}\left(x,t\right)=-\nabla \times E\left(x,t\right)$, showing the consistency with Maxwell’s equations. In a similar fashion, the Heisenberg equation of motion for the dynamical variable $X_{\omega }$ leads to the second-order differential equation

(10) $$\begin{array}{c} {\ddot{X}}_{\omega }\left(x,t\right)=-\omega^{2}X_{\omega }\left(x,t\right)+\mathrm{sgn}\left[\varepsilon_{I}\left(\omega \right)\right]g\left(x,\omega \right)E\left(x,t\right),\; \end{array}$$

which has the formal solution

(11) $$\begin{array}{ccc} X_{\omega }\left(x,t\right) & = & \left({\dot{X}}_{\omega }\left(x,0\right)\frac{\sin\omega t}{\omega }+X_{\omega }\left(x,0\right)\cos\omega t\right)\\ & & +g\left(x,\omega \right)\mathrm{sgn}\left[\varepsilon_{I}\left(x,\omega \right)\right]\int_{0}^{t}dt^{\prime }\frac{\sin\omega (t-t^{\prime })}{\omega }E\left(x,t^{\prime }\right). \end{array}$$

In classical electrodynamics, one would typically assume the corresponding initial fields ${\dot{X}}_{\omega }\left(x,0\right)$ and $X_{\omega }\left(x,0\right)$ to vanish, which is something that one should not do for the initial quantum operators in Equation (11), if only because this would violate their commutation relations. It is these initial-operator terms in Equation (11) that describe quantum noise, as we shall see shortly.

To facilitate our further calculations, let us introduce the annihilation operator

(12) $$d_{j}\left(x,\omega ,t\right)=\frac{1}{\sqrt{2\hbar \omega }}\left[-i\omega X_{\omega ,j}\left(x,t\right)+\;Q_{\omega ,j}\left(x,t\right)\right],$$

where j=1,2,3 labels the three orthogonal spatial directions. Their commutation relations follow immediately from Equation (7),

(13) $$\begin{array}{c} \left[d_{j}\left(x,\omega ,t\right),d_{j^{\prime }}^{†}\left(x^{\prime },\omega^{\prime },t\right)\right]=\;\delta_{jj^{\prime }}\;\delta \left(\omega -\omega^{\prime }\right)\delta^{3}\left(x-x^{\prime }\right).\;\;\;\;\;\;\; \end{array}$$

Now, by inverting the relations (12) and substituting the result into Equation (3), the polarization field of the medium can be written in terms of creation and annihilation operators as

(14) $$\begin{array}{c} P\left(x,t\right)=\varepsilon_{0}\int_{0}^{\infty }dt^{\prime }\;\chi \left(x,t-t^{\prime }\right)E\left(x,t^{\prime }\right)+P^{N}\left(x,t\right).\;\;\;\;\; \end{array}$$

Here, the time-dependent susceptibility is defined as

(15) $$\chi \left(x,t\right)=\frac{\Theta (t)}{\varepsilon_{0}}\int_{0}^{\infty }d\omega \;\mathrm{sgn}\left[\varepsilon_{I}\left(x,\omega \right)\right]g^{2}\left(x,\omega \right)\frac{\sin\omega t}{\omega },$$

which is a causal response function because of the step function Θ(t). After Fourier transformation, the susceptibility becomes

(16) $$\chi \left(x,\omega \right)=\frac{1}{\varepsilon_{0}}\int_{0}^{\infty }d\omega^{\prime }\frac{g^{2}\left(x,\omega^{\prime }\right)\;\mathrm{sgn}\left[\varepsilon_{I}\left(x,\omega^{\prime }\right)\right]}{\omega^{\prime 2}-{\left(\omega +i0^{+}\right)}^{2}}.$$

The field $P^{N}\left(x,t\right)$ in Equation (14) is the electric polarization noise density that is inevitably associated with absorption and amplification inside the medium. As in the phenomenological method of Refs. [70,71], we can separate this noise operator into positive- and negative-frequency parts $P^{N}=P^{N(+)}+P^{N(-)}$ with $P^{N(-)}={\left[P^{N(+)}\right]}^{†}$, where

(17) $$\begin{array}{c} P_{i}^{N(+)}\left(x,t\right)=i\int_{0}^{\infty }d\omega \;\sqrt{\frac{\hbar \varepsilon_{0}\left|\varepsilon_{I}\left(x,\omega \right)\right|}{\pi }}f_{i}\left(x,\omega \right)e^{-i\omega t},\;\;\;\;\;\;\;\;\; \end{array}$$

in terms of the operator $f_{i}\left(x,\omega \right)$ that has the form $d_{i}\left(x,\omega ,0\right)\Theta \left[\varepsilon_{I}\left(x,\omega \right)\right]+$ $d_{i}^{†}\left(x,\omega ,0\right)\Theta \left[-\varepsilon_{I}\left(x,\omega \right)\right]$. This noise operator is indeed expressed in terms of material operators at the initial time t=0, as anticipated. If we now take the time derivative of Maxwell’s equations in Equation (9) and insert Equation (14), then we obtain the frequency-domain wave equation for the positive-frequency part of the vector potential

(18) $$\begin{array}{ccc} & & \nabla \times \nabla \times A^{\left(+\right)}-\frac{\omega^{2}}{c^{2}}\varepsilon A^{\left(+\right)}=-i\mu_{0}\omega P^{N(+)}, \end{array}$$

where the electric permittivity ε(x,ω)=1+χ(x,ω) satisfies the Kramers–Kronig relations. Furthermore, the noise operator $P^{N(+)}\left(x,\omega \right)$ in the wave Equation (18) plays the role of a Langevin force associated with the quantum noise sources in the dielectric. Equation (18) can be solved as

(19) $$\begin{array}{ccc} A^{\left(+\right)}\left(x,t\right) & = & \frac{-i\mu_{0}}{\sqrt{2\pi }}\int_{0}^{\infty }d\omega \;\omega \int d^{3}x^{\prime }G\left(x,x^{\prime },\omega \right)\cdot P^{N(+)}\left(x^{\prime },\omega \right)\;e^{-i\omega t}\\ & = & \int_{0}^{\infty }d\omega \int d^{3}x^{\prime }\;\sqrt{\frac{\hbar \mu_{0}\omega^{2}\left|\varepsilon_{I}\left(x^{\prime },\omega \right)\right|}{\pi c^{2}}}G\left(x,x^{\prime },\omega \right)\cdot f\left(x^{\prime },\omega \right)e^{-i\omega t}, \end{array}$$

where $G(x,x^{\prime },\omega )$ is the classical causal Green function (a tensor) which is defined by

(20) $$\begin{array}{ccc} & & \left[\nabla \times \nabla \times -\frac{\omega^{2}}{c^{2}}\varepsilon \left(x,\omega \right)\right]G\left(x,x^{\prime },\omega \right)=\delta^{3}\left(x-x^{\prime }\right)1_{3}.\;\;\;\;\;\; \end{array}$$

From our Lagrangian theory, we, thus, arrive at the following more practical quantum theory of light, also known as Macroscopic QED, for an arbitrary inhomogeneous medium with both loss and gain: given a dielectric function ε(x,ω), compute the classical Green function (20) and use this to determine the vector potential (19). With Equation (6a) and Maxwell’s equations, all other electromagnetic field operators can then also be determined. This macroscopic QED formalism agrees with the one used in Ref. [79] and has broad applicability. For example, it could be used to generalize multi-emitter nanophotonics theories [80,81,82] to situations where background media can have loss but also gain. Here, instead, we will use it to derive quantum effective-medium theories for metamaterials.

## 3. Input–Output Operator Relations for Planar Dielectrics

**Figure 1:**
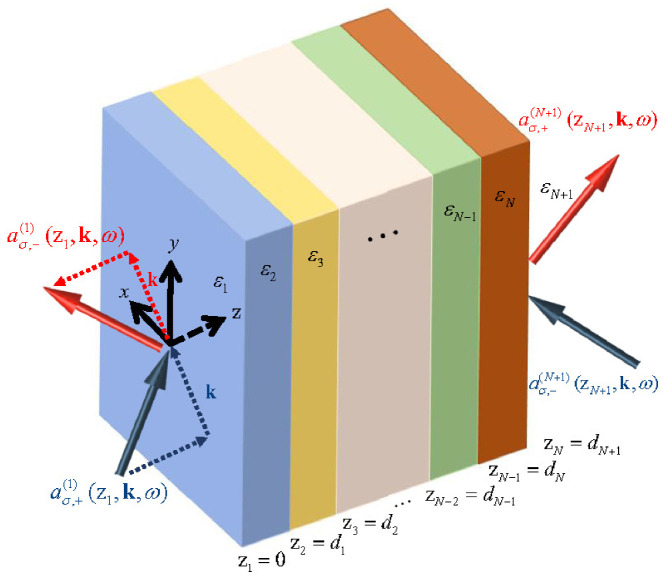
(Color online) Sketch of the planar dielectric medium with permittivity $\varepsilon_{j}\left(\omega \right)$ and thickness $d_{j}$ of the jth layer. The arrows denote incoming and outgoing fields. Additionally shown are the corresponding annihilation operators used in the definitions of the electric-field operator Equation (22).

Let us now specify that the dielectric medium with loss and/or gain is a planar dielectric for which the dielectric function ε(x,ω) varies in a step-wise fashion in the *z* direction, as depicted in Figure 1. The main goal of this paper is to propose and test effective-medium theories in quantum optics. The test consists of a comparison between an exact formalism for quantum optics in multilayer media on the one hand, and effective descriptions for planar metamaterials as “effectively homogeneous” on the other. Van der Waals heterostructures with weak electronic interlayer interactions are examples of planar nanomaterials that can be described using transfer-matrix methods and to which our multilayer formalism applies [83,84]. Here, we derive the exact multilayer formalism, which we compare with the effective description in Section 4.

We look for a quantum optical input–output relation that can describe the action of a lossy and/or linearly amplifying multilayer medium on an arbitrary quantum state of light incoming from an arbitrary direction with either *s*- or *p*-polarization, see Figure 1. This will be a gain-and-loss-in-3D generalization of the 1D formalism by Gruner et al., who studied the QED only of lossy planar dielectrics [67]. The incoming light constitutes an external light source, while quantum noise photons originating from lossy and especially from the amplifying layers are an internal source of light. The combination of both sources determines the quantum state of light that leaves the metamaterial. The sought input–output relation will reflect this.

We will derive the input–output relations by studying how the expressions for the electric-field operator at different spatial positions are related. As is convenient for multilayer media, we introduce the transverse spatial Fourier transform in the two directions of translational invariance. The electric-field operator in layer *j* becomes

(21) $$\begin{array}{c} E^{\left(j\right)}\left(x,t\right)=\frac{1}{{\left(2\pi \right)}^{3/2}}\int d^{2}k\int d\omega \left[e^{i(k\cdot \rho -\omega t)}\;E^{\left(j\right)}\left(z,k,\omega \right)+h.c.\right], \end{array}$$

where k is a two-dimensional vector in the x,y-subspace and ρ=(x,y). Here, the two-dimensional Fourier components of the electric-field operator are

$$\begin{array}{c} E^{\left(j\right)}\left(z,k,\omega \right)=\sum_{\sigma =s,p}\left[\;E_{\sigma ,+}^{\left(j\right)}\left(z,k,\omega \right)e_{\sigma ,+}^{\left(j\right)}\left(k\right)+E_{\sigma ,-}^{\left(j\right)}\left(z,k,\omega \right)e_{\sigma ,-}^{\left(j\right)}\left(k\right)\right], \end{array}$$

associated with light propagation both to the right (+) and left (−). We write these in terms of amplitude operators as

(22) $$\begin{array}{c} E_{\sigma ,\pm }^{\left(j\right)}\left(z,k,\omega \right)=\frac{i\omega }{\beta_{j}\;c}\sqrt{\frac{\hbar \beta_{j}^{\prime }}{2\varepsilon_{0}}}\;e^{\pm i\beta_{j}^{\prime }z}\;a_{\sigma ,\pm }^{\left(j\right)}\left(z,k,\omega \right). \end{array}$$

Since the properties of the amplitude operators $a_{\sigma ,\pm }^{\left(j\right)}\left(z,k,\omega \right)$ are still unspecified, at this point we only have given definitions. This changes by invoking the central result from the previous section, namely, that all Maxwell field operators, including the electric field, can be described in terms of the classical Green function. It comes in handy that we already derived the Green function of a multilayer medium with both lossy and amplifying layers in Ref. [76], as a generalization of the result by Tomaš for lossy dielectric multilayers [85]. In Appendix A, we give the (lengthy) explicit expression for the Green function, and discuss some subtle issues that arise for amplifying layers only.

Based on the explicit expression for the Green function, we show in Appendix B that in every separate layer the associated amplitude operators are bosonic operators that satisfy Langevin equations. Appendix B also contains the commutation relations of these amplitude operators. These properties characterize the amplitude operators in every layer separately. We note in passing that for frequencies ω far from the resonances of the medium, an ordinary normal-mode expansion for the electric-field operator is recovered: when gain and loss may be disregarded, i.e., in the limit $\varepsilon_{I,\;j}\left(\omega \right)\to 0$, the operators $a_{\sigma ,\pm }^{\left(j\right)}\left(z,k,\omega \right)$ become mode operators independent of *z*.

Knowing the amplitude operators in every layer separately is not enough, though. We can only find input–output relations by identifying relations between the amplitude operators in the various layers, and combining these to relate operators on opposite ends of the multilayer. In Appendix C, we derive this recursive procedure in three steps: first, within each layer *j*, we relate the amplitude operators on the extreme left and right to each other, i.e., at the positions $z=z_{j-1}$ and at $z=z_{j}$. Second, we relate the operators in neighboring layers across an interface, again based on the knowledge of the multilayer Green function. In the third step, by making repeated use of the previous two steps, we can relate the amplitude operators $a_{\sigma ,-}^{\left(1\right)}\left(z,k,\omega \right)$ and $a_{\sigma ,+}^{\left(N+1\right)}\left(z,k,\omega \right)$ for the outgoing fields to the left and right of the multilayer, respectively, to the operators of the corresponding incoming fields, $a_{\sigma ,+}^{\left(1\right)}\left(z,k,\omega \right)$ and $a_{\sigma ,-}^{\left(N+1\right)}\left(z,k,\omega \right)$, and to the noise amplitude operators $c_{\sigma ,\pm }^{\left(j\right)}\left(z,k,\omega \right)$ of each layer, as defined in Equation (A11). The sought input–output relation for amplitude operators is, thereby, obtained as

(23) $$\left(\begin{array}{c} a_{\sigma ,-}^{\left(1\right)}\left(z_{1}\right)\\ a_{\sigma ,+}^{\left(N+1\right)}\left(z_{N}\right) \end{array}\right)=T_{\sigma }\left(\begin{array}{c} a_{\sigma ,+}^{\left(1\right)}\left(z_{1}\right)\\ a_{\sigma ,-}^{\left(N+1\right)}\left(z_{N}\right) \end{array}\right)+\left(\begin{array}{c} F_{\sigma ,-}\\ F_{\sigma ,+} \end{array}\right),$$

where we suppressed the (k,ω)-dependence, and where the quantum noise originating from all layers with either loss or gain is given by

(24) $$\left(\begin{array}{c} F_{\sigma ,-}\\ F_{\sigma ,+} \end{array}\right)=D_{\sigma }^{\left(2\right)}\left(\begin{array}{c} c_{\sigma ,+}^{\left(2\right)}\\ c_{\sigma ,-}^{\left(2\right)} \end{array}\right)+\cdots +D_{\sigma }^{\left(N\right)}\left(\begin{array}{c} c_{\sigma ,+}^{\left(N\right)}\\ c_{\sigma ,-}^{\left(N\right)} \end{array}\right),$$

in which the coefficient matrices $T_{\sigma }$ and $D_{\sigma }$ are given by

(25a) $$T_{\sigma }=A_{\sigma \;22}^{-1}\left(\begin{array}{cc} -A_{\sigma 21}\;\; & 1\\ A_{\sigma 11}\;A_{\sigma 22}-A_{\sigma 12}\;A_{\sigma 21}\;\; & A_{\sigma 12} \end{array}\right),$$

(25b) $$\begin{array}{ccc} & & D_{\sigma }^{\left(j\;\right)}=A_{\sigma \;22}^{-1}\left(\begin{array}{cc} -B_{\sigma 21}^{\left(j\;\right)}\;\; & -B_{\sigma 22}^{\left(j\;\right)}\\ B_{\sigma 11}^{\left(j\;\right)}\;A_{\sigma 22}-A_{\sigma 12}\;B_{\sigma 21}^{\left(j\;\right)}\;\; & B_{\sigma 12}^{\left(j\;\right)}\;A_{\sigma 22}-A_{\sigma 12}\;B_{\sigma 22}^{\left(j\;\right)} \end{array}\right). \end{array}$$

Here, the matrices $B_{\sigma }^{\left(j\;\right)}$ satisfy the recursion relations $B_{\sigma }^{\left(k-1\;\right)}=B_{\sigma }^{\left(k\;\right)}\cdot R_{\sigma }^{\left(k\;\right)}\cdot S_{\sigma }^{\left(k-1\;\right)}$ and $B_{\sigma }^{\left(N\;\right)}=S_{\sigma }^{\left(N\;\right)}$, with k=3,4,…,N, and $A_{\sigma }=B_{\sigma }^{\left(2\;\right)}\cdot R_{\sigma }^{\left(2\;\right)}\cdot S_{\sigma }^{\left(1\;\right)}$. The multiple transmissions and reflections of the incident light in the multilayer medium are described by the same transfer matrices $T_{\sigma }$ as in classical optics. By contrast, the matrix elements $D_{\sigma }^{\left(j\;\right)}$ have no classical analogues, since they describe the propagation of quantum noise that originates from layer *j*.

The general input–output relation (23) for obliquely incident light on multilayer metamaterials with gain and loss reduces to the one that we used in our Ref. [59] for normal incidence (k=0). For purely passive multilayers, the latter input–output relation, in turn, reduces to the one in Ref. [67].

It is useful to know the commutation relations of the input and output operators. We consider the input operators first. Using the relations (A9) and the commutation relation (A8), we find that the input operators satisfy the commutation relations

(26a) $$\begin{array}{c} \left[a_{\sigma ,+}^{\left(1\right)}\left(z,k,\omega \right),a_{\sigma^{\prime },+}^{(1)\;†}\left(z^{\prime },k^{\prime },\omega^{\prime }\right)\right]=\varrho_{\sigma ,+}^{\left(1\right)}\;e^{-\beta^{\prime \prime }\left|z-z^{\prime }\right|}\mathrm{sgn}\left[\varepsilon_{I,\;1}\left(\omega \right)\right]\;\delta_{\sigma \sigma^{\prime }}\delta \left(\omega -\omega^{\prime }\right)\delta \left(k-k^{\prime }\right), \end{array}$$

and an analogous relation holds for the operators in the other outer layer labeled N+1 on the opposite size of the metamaterial, see Figure 1. It also follows that input operators of different outer layers commute,

(26b) $$\left[a_{\sigma ,+}^{\left(1\right)}\left(z,k,\omega \right),a_{\sigma^{\prime },-}^{(N+1)\;†}\left(z^{\prime },k^{\prime },\omega^{\prime }\right)\right]=0,$$

as expected for these independent input channels. We will not spell out the analogous commutation relations for the output amplitude operators $a_{\sigma ,-}^{\left(1\right)}\left(z,k,\omega \right)$ and $a_{\sigma ,+}^{\left(N+1\right)}\left(z,k,\omega \right)$, but they can be derived by applying the input–output relation (23) and the commutation relation (26a).

Indeed, the input–output relation (23), together with the commutation relations (26a), contain all information necessary to transform an arbitrary function of the input-field operators into the corresponding function of the output-field operators. In particular, it enables one to express arbitrary moments and correlations of the outgoing fields in terms of those of the incoming fields and the quantum-noise excitations in the multilayers.

## 4. Quantum Optical Effective-Index (QOEI) Theory

In Section 3, the problem was solved of how the output fields of multilayer media with both loss and gain depend on the input. This solution also holds for layered metamaterials: periodic multilayer media with unit cells much smaller than an optical wavelength. However, for these layered metamaterials, one can hope that a simpler, effective description as a homogeneous medium is also possible, here in quantum optics just like it is known to be possible in classical optics. Thus, we look for the effective index as well as the effective quantum noise of layered metamaterials.

The effective index in quantum optics is the same as in classical optics and can be determined using the same methods. We will focus on metamaterials with strongly subwavelength unit cells. This allows unique effective indices to be identified, since all methods to obtain them give practically the same values. By thus circumventing discussions on the level of classical effective-medium theory that can be found elsewhere, we can focus on the new quantum optical aspects.

Classical effective-medium theories are our starting point [11,12,13,14,15,17,18]. We will use and compare two established methods to determine effective indices. First, the scattering method by Smith and co-workers [11,12,18] boils down to finding the effective index of a homogeneous medium that mimics best the transmission and reflection off the metamaterial. Second, we use the dispersion method, where effective parameters are obtained from the small-(k,ω) Taylor expansion of the known dispersion relation of periodic multilayer structures. We briefly present both methods in Appendix D.

In this section, we present a quantum optical effective-index (QOEI) theory for three-dimensional light propagation in layered metamaterials, thereby generalizing the effective-index theory of Ref. [59] to arbitrary propagation directions and for two polarizations. By an effective-index theory, we mean a theory that describes the metamaterial entirely in terms of its effective index. In that sense, it does not differ from the usual effective theory in classical optics. However, it differs because quantum noise is also described. The crucial assumption of QOEI theory is that also the quantum noise of the metamaterial can effectively be described solely in terms of its effective index. This is the simplest quantum optical effective theory. For normal incidence, it was shown in Ref. [59] to be accurate for the important class of passive metamaterials. That is enough motivation to now generalize it for arbitrary angles of incidence and for both *s*- and *p*-polarizations. However, for loss-compensated media, QOEI theory was shown to fail [59]. In Section 5, we introduce a more generally valid effective theory for quantum optics, and in Section 6 we discuss its relation with QOEI theory.

*Output Operators of a Single Homogeneous Layer.—*We consider multilayer metamaterials surrounded by free space. Assume that we have used either the scattering or the dispersion method to determine the values for the effective dielectric tensor components for our multilayer structure. In addition, assume that in classical optics the entire structure can effectively be described as a single dielectric layer with effective permittivity function $\varepsilon_{\mathrm{eff},\;\sigma }$. Then we can also apply the elaborate quantum optical input-output formalism of Section 3 to that single effective layer. With the two planar interfaces of the homogenized slab located at $z_{1}=0$ and $z_{N}=L$, the input–output relation (23) for the single effective layer reduces to the simpler form

(27) $$\begin{array}{c} \left(\begin{array}{c} a_{\sigma ,-}^{\left(1\right)}\left(z_{1},k,\omega \right)\\ a_{\sigma ,+}^{\left(N+1\right)}\left(z_{N},k,\omega \right) \end{array}\right)=T_{\mathrm{eff},\;\sigma }\left(\begin{array}{c} a_{\sigma ,+}^{\left(1\right)}\left(z_{1},k,\omega \right)\\ a_{\sigma ,-}^{\left(N+1\right)}\left(z_{N},k,\omega \right) \end{array}\right)+\left(\begin{array}{c} F_{\mathrm{eff}\;\sigma ,-}\left(k,\omega \right)\\ F_{\mathrm{eff}\;\sigma ,+}\left(k,\omega \right) \end{array}\right), \end{array}$$

where according to Equation (25a), the matrix presentation $T_{\mathrm{eff},\;\sigma }$ is equal to

(28) $$\left(\begin{array}{cc} r_{\mathrm{eff},\;\sigma }\;\;\;\; & t_{\mathrm{eff},\;\sigma }\\ t_{\mathrm{eff},\;\sigma }\;\;\;\; & e^{-2i\beta_{0}L}\;r_{\mathrm{eff},\;\sigma } \end{array}\right),$$

and where the effective complex reflection and transmission amplitudes of the homogenized slab are given by the well-known classical expressions

(29a) $$\begin{array}{ccc} r_{\mathrm{eff},\;\sigma } & = & \frac{\left(\beta_{\mathrm{eff},\;\sigma }^{2}-\epsilon_{\sigma }\beta_{0}^{2}\right)\left(\exp\left[2i\beta_{\mathrm{eff},\;\sigma }L\right]-1\right)}{{\left(\beta_{\mathrm{eff},\;\sigma }+\epsilon_{\sigma }\beta_{0}\right)}^{2}-{\left(\beta_{\mathrm{eff},\;\sigma }-\epsilon_{\sigma }\beta_{0}\right)}^{2}\exp\left[2i\beta_{\mathrm{eff},\;\sigma }L\right]}, \end{array}$$

(29b) $$\begin{array}{ccc} t_{\mathrm{eff},\;\sigma } & = & \frac{4\epsilon_{\sigma }\beta_{0}\beta_{\mathrm{eff},\;\sigma }\exp\left[i\left(\beta_{\mathrm{eff},\;\sigma }-\beta_{0}\right)L\right]}{{\left(\beta_{\mathrm{eff},\;\sigma }+\epsilon_{\sigma }\beta_{0}\right)}^{2}-{\left(\beta_{\mathrm{eff},\;\sigma }-\epsilon_{\sigma }\beta_{0}\right)}^{2}\exp\left[2i\beta_{\mathrm{eff},\;\sigma }L\right]}.\;\;\;\;\;\;\;\;\;\;\;\; \end{array}$$

Here, $\epsilon_{s}=1$, $\epsilon_{p}=\varepsilon_{\mathrm{eff},\;p}$, and $\beta_{\mathrm{eff},\;\sigma }=\sqrt{\varepsilon_{\mathrm{eff},\;\sigma }\omega^{2}/c^{2}-k^{2}}$, in which $\varepsilon_{\mathrm{eff},\;p}$ depends on the angle of the incident light (for more details, see Appendix D). The effective noise operators $F_{\mathrm{eff},\;\sigma ,\pm }$ in Equation (27) have no classical analogues. In Appendix E, we give the expressions of the $F_{\mathrm{eff},\;\sigma ,\pm }$ in terms of fundamental bosonic noise operators, which lead to the commutation relations

(30a) $$\begin{array}{ccc} & & \;\left[F_{\mathrm{eff}\;\sigma ,\pm }\left(k,\omega \right),\;F_{\mathrm{eff}\;\sigma^{\prime },\pm }^{†}\left(k^{\prime },\omega^{\prime }\right)\right]=\left(1-\right|r_{\mathrm{eff},\;\sigma }{|}^{2}-|t_{\mathrm{eff},\;\sigma }{|}^{2})\;\delta_{\sigma \;\sigma^{\prime }}\delta \left(k-k^{\prime }\right)\delta \left(\omega -\omega^{\prime }\right), \end{array}$$

(30b) $$\begin{array}{ccc} & & \;\left[F_{\mathrm{eff}\;\sigma ,\pm }\left(k,\omega \right),F_{\mathrm{eff}\;\sigma^{\prime },\mp }^{†}\left(k^{\prime },\omega^{\prime }\right)\right]=-\left(r_{\mathrm{eff},\sigma }t_{\mathrm{eff},\sigma }^{*}+e^{2i\beta_{0}L}r_{\mathrm{eff},\sigma }^{*}t_{\mathrm{eff},\sigma }\right)\delta_{\sigma \sigma^{\prime }}\delta \left(k-k^{\prime }\right)\delta \left(\omega -\omega^{\prime }\right), \end{array}$$

in terms of the reflection and transmission amplitudes of Equation (29). The input operators of the effective slab satisfy the bosonic commutation relations

(31) $$\begin{array}{c} \left[a_{\sigma ,+}^{\left(1\right)}\left(k,\omega \right),\;a_{\sigma^{\prime },+}^{(1)\;†}\left(k^{\prime },\omega^{\prime }\right)\right]=\left[a_{\sigma ,-}^{\left(N+1\right)}\left(k,\omega \right),\;a_{\sigma^{\prime },-}^{(N+1)\;†}\left(k^{\prime },\omega^{\prime }\right)\right]=\delta_{\sigma \;\sigma^{\prime }}\delta \left(k-k^{\prime }\right)\delta \left(\omega -\omega^{\prime }\right), \end{array}$$

which indeed agrees with Equation (26a) for the general multilayer in Section 3 when assuming the outer layers to be free space. For the output-mode operators $a_{\sigma ,-}^{\left(1\right)}$ and $a_{\sigma ,+}^{\left(N+1\right)}$, the commutation relations can also be obtained, by combining Equations (30) and (31) with the input–output relations (27), giving

(32) $$\begin{array}{c} \left[a_{\sigma ,-}^{\left(1\right)}\left(k,\omega \right),\;a_{\sigma^{\prime },-}^{(1)\;†}\left(k^{\prime },\omega^{\prime }\right)\right]=\left[a_{\sigma ,+}^{\left(N+1\right)}\left(k,\omega \right),\;a_{\sigma^{\prime },+}^{(N+1)\;†}\left(k^{\prime },\omega^{\prime }\right)\right]=\delta_{\sigma \;\sigma^{\prime }}\delta \left(k-k^{\prime }\right)\delta \left(\omega -\omega^{\prime }\right). \end{array}$$

Therefore, despite the complex input–output relations, the output operators have the same simple standard commutation relations as the input operators, thanks to the less trivial commutation relations of the noise operators.

The quantum optical effective-index theory for planar metamaterials is hereby defined. A more complex but also more generally valid effective theory will be presented in Section 5. Two subsequent sections comprise test cases for both effective theories.

## 5. Quantum Optical Effective-Medium (QOEM) Theory

We will now derive a quantum optical effective-medium (QOEM) theory that does give accurate predictions for thee-dimensional light propagation in loss-compensated media. In contrast to QOEI theory, it is not an effective-index theory, because besides the effective index, another effective parameter will be needed. Our approach is to distill solely from a unit cell not only the usual $\beta_{\mathrm{eff},\;\sigma }$ but also an effective noise photon distribution $N_{\mathrm{eff},\;\sigma }\left(k,\omega ,T\right)$. The theory presented here is a generalization of Ref. [59], which is valid for normal incidence, to arbitrary angles of incidence and polarization.

Analogous to effective-index theory, we will again assume that there is an effective noise operator in the unit cell. However, unlike in effective-index theory, we will not try to define this operator, but rather determine the expectation value of its corresponding number operator. Analogous to what we will find in Equation (39) for the effective-index theory, we write the expectation value

(33) $$\begin{array}{ccc} & & {\langle F_{\sigma }^{†}\left(k,\omega \right)F_{\sigma^{\prime }}\left(k^{\prime },\omega^{\prime }\right)\rangle }_{\mathrm{QOEM}}=\delta_{\sigma \;\sigma^{\prime }}\delta \left(k-k^{\prime }\right)\delta \left(\omega -\omega^{\prime }\right)\\ & & \times \left\{N_{\mathrm{eff},\;\sigma }\left(k,\omega ,T\right)\Theta \left[\varepsilon_{\mathrm{unit},\mathrm{eff}\;I}\left(\omega \right)\right]-\left(N_{\mathrm{eff},\;\sigma }\left(k,\omega ,|T|\right)+1\right)\Theta \left[-\varepsilon_{\mathrm{unit},\mathrm{eff}\;I}\left(\omega \right)\right]\right\}\\ & & \times \left(1-|R_{\mathrm{unit},\mathrm{eff},\;\sigma }{|}^{2}-{\left|T_{\mathrm{unit},\mathrm{eff},\;\sigma }\right|}^{2}\right), \end{array}$$

in terms of the effective noise current density $N_{\mathrm{eff}}$, which we define shortly. The $R_{\mathrm{unit},\mathrm{eff},\;\sigma }$ and $T_{\mathrm{unit},\mathrm{eff},\;\sigma }$ are the (classical) reflection and transmission amplitudes of the entire unit cell. If the factor $\left(1-|R_{\mathrm{unit},\mathrm{eff},\;\sigma }{|}^{2}-|T_{\mathrm{unit},\mathrm{eff},\;\sigma }{|}^{2}\right)$ is positive then it quantifies the amount of net absorption in the unit cell, and, otherwise, the net amplification. The effective distribution $N_{\mathrm{eff}}$, in general, is not a thermal one, in contrast to the distribution for the effective-index theory as discussed in Equation (39) below, which features thermal distributions $N_{\mathrm{th}}$.

We fix ${\langle F^{†}F\rangle }_{\mathrm{QOEM}}$ of Equation (33) and, thereby, $N_{\mathrm{eff},\;\sigma }$ in three steps. First, we apply our general input-output theory of Section 3 to a single unit cell of the metamaterial. Second, we require that the expectation value ${\langle F^{†}F\rangle }_{\mathrm{QOEM}}$ coincides with the corresponding unit-cell-averaged noise expectation value of the general multilayer theory. Third, we make use of our assumption that the unit cell of the metamaterial is much thinner than an optical wavelength, so we can Taylor expand the results from multilayer theory to first order in the layer thicknesses $d_{a,b}$ and obtain

(34) $$\begin{array}{c} \;{\langle F_{\sigma }^{†}\left(k,\omega \right)F_{\sigma^{\prime }}\left(k^{\prime },\omega^{\prime }\right)\rangle }_{\mathrm{QOEM}}=\sum_{j=a,b}\frac{d_{j}\;\left|\varepsilon_{j,I}\right|\;\omega^{2}\;K_{j,\sigma }\left(\theta \right)}{c^{2}\beta_{0}}N_{\mathrm{th}}\left(\omega ,\right|T_{j}\left|\right)\;\delta_{\sigma \;\sigma^{\prime }}\delta \left(k-k^{\prime }\right)\delta \left(\omega -\omega^{\prime }\right), \end{array}$$

where for *s*-polarization the factor $K_{j,s}\left(\theta \right)$ simply equals unity while $K_{j,p}\left(\theta \right)=\cos^{2}\theta +\sin^{2}\theta /{\left|\varepsilon_{j}\right|}^{2}$ for *p*-polarized light. Now we have two expressions for ${\langle F^{†}F\rangle }_{\mathrm{QOEM}}$, namely, Equations (33) and (34). By equating these two, Taylor approximating also the net gain or loss factor $\left(1-|R_{\mathrm{unit},\mathrm{eff},\;\sigma }{|}^{2}-{\left|T_{\mathrm{unit},\mathrm{eff},\;\sigma }\right|}^{2}\right)$ of Equation (33) to first order in the unit cell thickness $d=d_{a}+d_{b}$, and solving for $N_{\mathrm{eff},\;\sigma }\left(k,\omega ,T\right)$, we obtain as a main result the effective noise photon distribution

(35) $$\begin{array}{c} N_{\mathrm{eff},\;\sigma }\left(k,\omega ,T\right)=\left\{\begin{array}{c} \sum_{j=a,b}\eta_{j,\sigma }\left[N_{\mathrm{th}}\left(\omega ,T_{j}\right)\right]\\ -1+\sum_{j=a,b}\eta_{j,\sigma }[N_{\mathrm{th}}\left(\omega ,\right|T_{j}\left|)+1\right]\\ -\frac{1}{2}+\frac{1}{2}\sum_{j=l,g}\eta_{j,\sigma }[2N_{\mathrm{th}}\left(\omega ,\right|T_{j}\left|)+1\right] \end{array}\right. \end{array}$$

which correspond, from top to bottom, to loss-loss, gain-gain, and loss-compensated metamaterials. This $N_{\mathrm{eff}}$, thus, depends on the same variables as the classical effective parameter $\beta_{\mathrm{eff}}$: on the angle of incidence, on the polarization of the input state, as well as on the dielectric parameters of the unit cell via

(36) $$\begin{array}{c} \eta_{j,\sigma }\left(\theta \right)=p_{j}\;\frac{K_{j,\sigma }\left(\theta \right)}{K_{\mathrm{eff},\sigma }\left(\theta \right)}\left|\frac{\varepsilon_{j,\;I}\left(\omega \right)}{\varepsilon_{\mathrm{eff}\;\sigma ,\;I}\left(\omega \right)}\right|, \end{array}$$

where the $p_{j}=d_{j}/d$ are the volume fractions of the layers, and the factor $K_{\mathrm{eff},\;\sigma }\left(\theta \right)$ equals $K_{j,\;\sigma }\left(\theta \right)$ with $\varepsilon_{j}$ replaced by $\varepsilon_{\mathrm{eff},\;\sigma }$. We allowed the two types of layers of the unit cell to be at different temperatures. Generalizations to more than two layers per unit cell are straightforward.

Let us first apply this QOEM theory to loss-compensated metamaterials. To gain some intuition about the new effective parameter $N_{\mathrm{eff},\;\sigma }$, notice that from Equations (35) and (36) it follows that $N_{\mathrm{eff}}$ grows when loss in the metamaterial is more exactly compensated by gain (smaller $\varepsilon_{\mathrm{eff},\;\sigma }$) or when the same value $\varepsilon_{\mathrm{eff},\;\sigma }$ results from compensating more loss by more gain (i.e., with $\left|\varepsilon_{a,\;I}\left(\omega \right)\right|)$ and $\left|\varepsilon_{a,\;I}\left(\omega \right)\right|)$ both larger). This means that for metamaterials with more effective loss compensation, it becomes increasingly important to use $N_{\mathrm{eff},\;\sigma }$ as an additional effective-medium parameter instead of $N_{\mathrm{th}}$. We will illustrate this in Section 7.

Let us now show that our new QOEM theory indeed reduces to the one of Ref. [59] in case of light propagation perpendicular to the interface, i.e., for k=0. In that case, the parameter $K_{j,\sigma }\left(\theta \right)$ in Equation (34) tends to unity for both polarizations. This, in turn, implies that the parameter $\eta_{j,\sigma }\left(\theta \right)$ defined in Equation (36) tends to $p_{j}\left|\varepsilon_{j,\;I}\left(\omega \right)/\varepsilon_{\mathrm{eff}\;,I}\left(\omega \right)\right|$, since at normal incidence the two polarizations are degenerate and $\varepsilon_{\mathrm{eff},\;s}=\varepsilon_{\mathrm{eff},\;p}=\varepsilon_{\mathrm{eff}}$. This, indeed, agrees with Ref. [59], where we showed that for normal incidence the quantum optical effective-medium theory gave accurate predictions for loss-compensated, loss-loss as well as gain-gain metamaterials.

## 6. When Does QOEM Coincide with QOEI Theory?

Do we also need the QOEM theory for loss-loss or gain-gain metamaterials, or does the simpler QOEI theory suffice? The QOEI theory features only one temperature for the metamaterial. If the two layers within the unit cell are somehow kept at different temperatures, which is not easy to realize, then we would need QOEM instead of QOEI theory. However, if the entire unit cell is kept at the same temperature, then for light propagation normal to the layers we found in Ref. [59] that QOEM theory reduces to the QOEI theory, i.e., $N_{\mathrm{eff}}$ becomes equal to the thermal noise photon distribution $N_{\mathrm{th}}$. Is this also true for oblique incidence?

**Figure 2:**
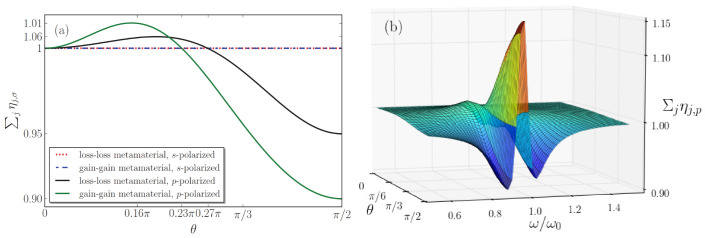
(Color online) (**a**) The sum $\Sigma_{j=a,b}\eta_{j,\sigma }$ is shown as a function of the angle of incidence θ for *s*-polarized component of input light impinging on the loss-loss (dotted red line) and the gain-gain (dash-dotted blue line) multilayers, and *p*-polarized component of input light incident on the loss-loss (solid black line) and the gain-gain (solid green line) multilayers. (**b**) The sum $\Sigma_{j=a,b}\eta_{j,p}$ is shown as a function of the angle of incidence θ and of the dimensionless frequency $\omega /\omega_{0}$ for *p*-polarized component of input light impinging on a gain-gain multilayer. The multilayer metamaterial with geometry of Figure 6 has alternating layers with equal thickness $d_{a,b}\omega_{0}/c=0.1$, with dielectric parameters in Equation (40): $\omega_{p_{a}}/\omega_{0}=0.3$, $\omega_{p_{b}}/\omega_{0}=0.1$ and $\gamma_{a,b}/\omega_{0}=0.1$. In panel (**a**), we choose $\omega /\omega_{0}=0.9$.

Let us consider *s*-polarized light first, for loss-loss and gain-gain metamaterials at a uniform temperature ($T_{a}=T_{b}$). This means, technically, that the thermal distributions in Equation (35) can be moved in front of the summation, and the remaining summation is $\sum_{j=a,b}\eta_{j,s}\left(\theta \right)$. Since in $\eta_{j,s}\left(\theta \right)$, as defined in Equation (36), the fractions $K_{j,s}\left(\theta \right)/K_{\mathrm{eff},s}\left(\theta \right)$ are equal to unity, the sum $\sum_{j=a,b}\eta_{j,s}\left(\theta \right)$ becomes $\sum_{j=a,b}p_{j}\left|\varepsilon_{j,\;I}\left(\omega \right)/\varepsilon_{\mathrm{eff},\;I}\left(\omega \right)\right|$, which is angle-independent. In Ref. [59], we also pointed out that for normal incidence the sum $\sum_{j=a,b}p_{j}\left|\varepsilon_{j,\;I}\left(\omega \right)/\varepsilon_{\mathrm{eff},\;I}\left(\omega \right)\right|$ adds up to unity for loss-loss and gain-gain metamaterials (whereas the sum is always larger than unity for loss-compensated (gain-loss) metamaterials). Therefore, now we find that for *s*-polarized light, the same relations even hold for arbitrary angles of incidence, as illustrated by the horizontal line in Figure 2a. As a consequence, we find that for *s*-polarized light incident on loss-loss or gain-gain MMs, QOEM theory coincides with QOEI theory.

How about *p*-polarized light then, does $N_{\mathrm{eff}}$ also reduce to the thermal distribution for loss-loss and gain-gain metamaterials? For MMs kept at a uniform temperature, the thermal factor in the summation of Equation (35) can again be put in front, so that the summation reduces to $\sum_{j=a,b}\eta_{j,p}\left(\theta \right)$. However, for *p*-polarized light, the fractions $K_{j,p}\left(\theta \right)/K_{\mathrm{eff},p}\left(\theta \right)$ do not give unity, and, hence, the sum $\sum_{j=a,b}\eta_{j,p}\left(\theta \right)$ in general does not add up to unity. As a consequence, for *p*-polarized light incident on loss-loss or gain-gain multilayers, the $N_{\mathrm{eff}}$ of Equation (35), in general, does not reduce to the thermal distribution, and, consequently, QOEM theory does *not* coincide with QOEI theory.

We were especially surprised to find that QOEM theory does not coincide exactly with QOEI theory for all passive metamaterials. In Figure 2, we study numerically how much the two theories differ. The closer the sum $\sum_{j}\eta_{j,p}$ comes to unity, the closer QOEM theory comes to the QOEI theory. This sum is thus a measure for the “distance” between the two theories, and it depends on the dielectric parameters of the unit cell. The angle dependence of this sum is illustrated in Figure 2a. The deviations of $N_{\mathrm{eff}}$ from a thermal distribution are within ten percent for the specific dielectric parameters chosen. $N_{\mathrm{eff}}$ becomes larger (smaller) than the thermal distribution $N_{\mathrm{th}}$ for angles of incidence smaller (larger) than 0.27π for the loss-loss metamaterial, and for the gain-gain MM the critical angle occurs at 0.23π.

It also depends on frequency how well QOEM theory can be approximated by QOEI theory. In Figure 2b, we depict the sum $\Sigma_{j}\eta_{j,p}$ as a function of both the angle of incidence θ and of the dimensionless frequency $\omega /\omega_{0}$, for the same gain-gain MM as in Figure 2a. Here, the sum $\sum_{j}\eta_{j,p}$ has a maximal deviation from unity of around 15 percent. We find similar non-negligible frequency and angle dependence (not shown) for the loss-loss multilayer of Figure 2a.

After these theoretical comparisons of the two effective-medium theories, in the following sections we will test their accuracies in predicting two experimentally measurable quantities.

## 7. First Test: Power Spectra

As a first test and comparison of the QOEI and QOEM theories with the exact multilayer theory, we will now study the output intensities of light due to spontaneously emitted photons. If atoms that make up the metamaterial are excited, either thermally or because of external pumping, then they can decay spontaneously. This is a known noise source in lasers, which is typically overlooked for metamaterials. There is a variety of different quantum definitions of the power spectrum in the literature [86]. Here, we choose the quantum generalization of the classical definition of the energy spectrum for the case of a stationary field [86]. Just like its classical counterpart, it is directly related to observables in light detection experiments. For sufficiently small pass-band widths of the spectral apparatus, the power spectrum S(x,ω) of the light emitted on the right-hand side of our multilayer metamaterial of Figure 1 is given by

(37) $$\begin{array}{c} S\left(x,\omega \right)=\lim_{T\to \infty }\frac{1}{2\pi T}\overset{T/2}{\underset{-T/2}{\;\int \int \;}}dtdt^{\prime }\;e^{-i\omega (t-t^{\prime })}\langle E^{(N+1)-}\left(x,t\right)\cdot E^{(N+1)+}\left(x,t^{\prime }\right)\rangle , \end{array}$$

where ω is the operating frequency of the spectral apparatus, and *T* is the duration for which the detector is switched on. We insert the electric-field operators $E^{N+1(\pm )}$ of Equation (22).

In general, the power spectrum depends both on the incoming optical fields and on the quantum noise in the medium. Our goal is here to find out how well quantum optical effective-medium theories describe the amount of quantum noise photons that contribute to photon-counting measurements. In this section, we will, therefore, study output intensities in the absence of any optical input signal, in other words, all optical incoming fields are assumed to be in the vacuum state |0〉. In that case, all output photons are spontaneously emitted noise photons, or S(x,ω) is equal to

(38) $$\begin{array}{ccc} S_{\mathrm{Spon}}\left(\omega \right) & = & \sum_{\sigma }\int_{0}^{\pi /2}d\theta \;S_{\mathrm{Spon},\sigma }\left(\theta ,\omega \right)\\ & = & \frac{\hbar \omega^{2}}{8\pi^{2}\varepsilon_{0}c^{2}}\sum_{\sigma }\int dk\;\beta_{0}^{-1}\;\langle F_{\sigma ,+}^{†}\left(k,\omega \right)\;F_{\sigma ,+}\left(k,\omega \right)\rangle . \end{array}$$

Clearly, the power spectrum of the spontaneously emitted light depends on the quantum noise through the expectation value of $\langle F_{\sigma ,+}^{†}\left(k,\omega \right)\;F_{\sigma ,+}\left(k,\omega \right)\rangle$.

In the following, we will mostly consider power spectra at zero temperature. Absorbing layers do not emit thermal photons in that case, but amplifying layers have population inversion and their excited-state population can decay spontaneously. In our numerical examples, we will look at the polarization- and angle-dependent power spectrum $S_{\mathrm{Spon},\sigma }\left(\theta ,\omega \right)$ that was defined in terms of $\langle F_{\sigma ,+}^{†}\left(k,\omega \right)\;\;F_{\sigma^{\prime },+}\left(k^{\prime },\omega^{\prime }\right)\rangle$ in Equation (38), and where we assumed that only propagating modes reach the detector and, thus, restricted the Fourier integral to modes with |k|>ω/c.

What corresponding power spectrum does the QOEI theory of Section 4 predict? From the definitions (A18a) together with the commutation relation (30), the flux in noise photons emitted by the multilayer slab at a finite temperature *T* can, within the effective-index theory, be expressed in terms of the effective reflection and transmission amplitudes as

(39) $$\begin{array}{ccc} & & {\langle F_{\mathrm{eff}\;\sigma ,\pm }^{†}\left(k,\omega \right)\;F_{\mathrm{eff}\;\sigma^{\prime },\pm }\left(k^{\prime },\omega^{\prime }\right)\rangle }_{\mathrm{QOEI}}=\\ & & \left\{N_{\mathrm{th}}\left(\omega ,T\right)\Theta \left[\varepsilon_{\mathrm{eff},\;I}\left(\omega \right)\right]-\left(N_{\mathrm{th}}\left(\omega ,|T|\right)+1\right)\Theta \left[-\varepsilon_{\mathrm{eff},\;I}\left(\omega \right)\right]\right\}\\ & & \times (1-|r_{\mathrm{eff},\;\sigma }{|}^{2}-|t_{\mathrm{eff},\;\sigma }{|}^{2})\delta_{\sigma \;\sigma^{\prime }}\delta \left(k-k^{\prime }\right)\delta \left(\omega -\omega^{\prime }\right). \end{array}$$

Here, $k_{B}$ is the Boltzmann constant and *T* is the temperature, and $N_{\mathrm{th}}=1/(\exp\left[\hbar \omega /k_{B}T\right]$−1) is the thermal distribution of photon states at energy ℏω. Notice that this flux in noise photons in Equation (39) is always a non-negative quantity (as it should be): for media that are effectively absorbing at frequency ω, the $\varepsilon_{\mathrm{eff},\;I}\left(\omega \right)$ is positive and so is $\left(1-|r_{\mathrm{eff},\;\sigma }{|}^{2}-|t_{\mathrm{eff},\;\sigma }{|}^{2}\right)$, while for effectively amplifying media, both these quantities are negative. The QOEI power spectrum of the spontaneously emitted light (38) is now obtained by substituting Equations (39) and (29) into Equation (38).

To be specific, unless stated explicitly, below, in our numerical examples, we will assume the temperature to be zero Kelvin. Furthermore, we will assume that lossy and amplifying layers can be described by Lorentzian dielectric functions. A medium consisting of two-level atoms with a population $N_{\mathrm{up}}$ in the upper level and $N_{\mathrm{down}}$ in the lower level can near its resonance frequency $\omega_{0}$ be described by an electric permittivity of the Lorentzian form [70]

(40) $$\begin{array}{c} \varepsilon \left(\omega \right)=1+\left(\frac{N_{\mathrm{down}}-N_{\mathrm{up}}}{N_{\mathrm{down}}+N_{\mathrm{up}}}\right)\frac{\omega_{p}^{2}}{\omega_{0}^{2}-\omega^{2}-i\gamma \omega }. \end{array}$$

where $\omega_{p}$ is the coupling frequency, $\omega_{0}$ is the transverse resonance frequency, and γ is the dissipation and amplification parameters for lossy and amplifying layers, respectively. The population factor that occurs in the dielectric function (40) is positive for passive systems, $N_{\mathrm{down}}>N_{\mathrm{up}}$, but negative for optical gain that arises from population inversion in the medium, $N_{\mathrm{up}}>N_{\mathrm{down}}$. In addition, this factor can be written in terms of the thermal distribution $N_{\mathrm{th}}$ as ${\left[2N_{\mathrm{th}}\left(\omega ,T\right)+1\right]}^{-1}$ for lossy and as $[-2N_{\mathrm{th}}{\left(\omega ,|T|)-1\right]}^{-1}$ for amplifying layers.

### 7.1. Power Spectra of Loss-Compensated MM

**Figure 3:**
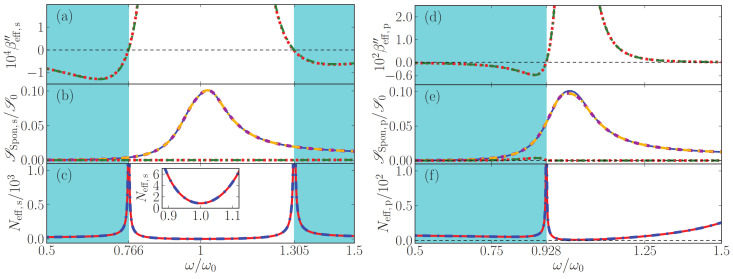
The power spectrum (A20) of the spontaneous emission of noise photons exiting a loss-compensated multilayer metamaterial at an angle of 30 degrees away from the normal, in units of $S_{0}=\hbar \omega_{0}^{3}/4\pi \varepsilon_{0}c^{3}$, due to spontaneous emission of noise photons within the loss-compensated multilayer metamaterial is sketched in Figure 6, at zero temperature. Left and right panels correspond to *s*- and *p*-polarized light, respectively. The amplifying and absorbing layers are described by the Lorentz model (Equation (40)), with parameters $\omega_{p_{a}}/\omega_{0}=0.3$, $\gamma_{a}/\omega_{0}=0.1$ for the lossy layers, and $\omega_{p_{b}}/\omega_{0}=0.25$, $\gamma_{b}/\omega_{0}=0.15$ for the layers with gain. We choose $d_{a,b}\omega_{0}/c=0.1$ and five unit cells. The parts (**a**,**d**) show the imaginary part of the normal wave-vector component $\beta_{\mathrm{eff}}$. In panels (**b**,**e**), the power spectrum of the noise photons predicted with the effective-index theories is compared to the exact multilayer calculation and the QOEM theory. For the effective-index theories, red dotted curves are obtained by inserting effective parameters based on Equation (A15a) into Equation (A20); the green dash-dotted lines correspond to the other procedure Equation (A17) to obtain effective parameters. Similarly, for QOEM theory (discussed in Section 5), the magenta dashed lines are produced with Equation (A15a), and the yellow dash-dotted curves with Equation (A17). Panels (**c**,**f**) show the effective noise current densities $N_{\mathrm{eff}}$ of Equation (35), in solid red lines as obtained using the effective index of Equation (A15a) and in dash-dotted blue curves as produced with the other procedure (Equation (A17)) to obtain the effective index.

**Figure 4:**
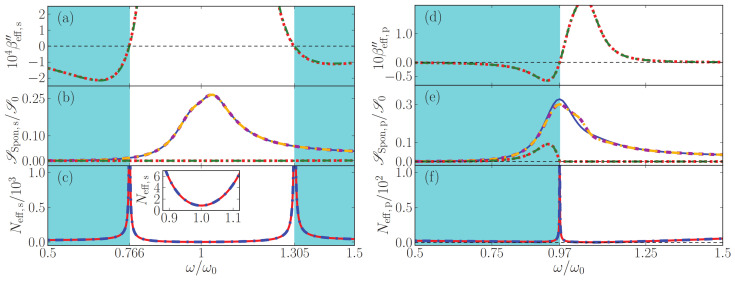
As in Figure 3 but now for light emission at an angle of 60 degrees with respect to the normal.

In Figure 3, we explore regions with net loss and net gain and the frequencies of exact loss compensation that separate them, and study the corresponding flux of noise photons and the effective noise photon distribution, all corresponding to an output angle of $30^{\circ }$. Left panels depict *s*- and right panels *p*-polarization. In Figure 4, we show the analogous results for an emission angle of $60^{\circ }$. (Some panels of the two figures will be explained below in Section 5).

Exact loss compensation occurs when the imaginary part of the normal-wave vector components $\beta_{\mathrm{eff}}$ vanishes. We show $\beta_{\mathrm{eff}}$ in panels (a) and (d) of Figure 3 and Figure 4, which also confirm that the two methods to retrieve effective parameters lead to nearly identical results.

For *s*-polarization, it follows from Equation (A17) that exact loss compensation occurs at angle-independent frequencies. A comparison of the panels (a) of Figure 3 and Figure 4 illustrates this, where, for the parameters chosen, exact loss compensation occurs at $0.766\omega_{0}$ and $1.305\omega_{0}$, net loss in the frequency range $0.766<\omega /\omega_{0}<1.305$ and net gain at elsewhere.

By contrast, for *p*-polarized light, exact loss compensation does depend on the angle of incidence, as again follows from Equation (A17) and as illustrated in Figure 3 and Figure 4: in Figure 3d, exact loss compensation occurs (only) at $\omega =0.928424\omega_{0}$, with net gain at smaller and net loss at higher frequencies. At sixty degrees, Figure 4d shows exact loss compensation at a slightly higher frequency.

In panels (b) for *s*-polarization and (e) for *p*-polarization, power spectra are displayed for spontaneously emitted light that exits the metamaterial at an angle of $30^{\circ }$ (Figure 3) and $60^{\circ }$ (Figure 4). Note that these angular power spectra are continuous also across frequencies of exact loss compensation, whereas the effective noise photon densities $N_{\mathrm{eff}}$ actually diverge at these specific frequencies, see panels (c) and (f).

For lossy homogeneous media at zero temperature, the flux of thermal noise photons vanishes, so the power spectrum of the outgoing noise photons vanishes. Effective-index theory predicts something else, namely, that no photons are emitted by *effectively* lossy loss-compensated metamaterials. This prediction is illustrated in panels (b) and (e) of Figure 3 and Figure 4, especially around $\omega_{0}$ for outgoing *s*-polarized light, and above $0.928424\omega_{0}$ for outgoing *p*-polarized light. By contrast, the full gain-loss multilayer calculation does predict the emission of noise photons at zero temperature, as the figures show. Thus, effective-index theory clearly fails for effectively lossy loss-compensated metamaterials. At exact loss compensation ($\varepsilon_{\mathrm{eff},\;I}=0$), by Equation (39), the effective-index theory predicts that the flux of noise photons vanishes, which the figures show is another failure of the effective-index theory.

For effectively amplifying loss-compensated metamaterials, effective-index theory does predict a finite flux of spontaneously emitted photons which grows with the effective gain, as is best visible in Figure 4d, where, for frequencies slightly below $0.97\omega_{0}$, much loss is slightly overcompensated by much gain. Again, the effective-index theory is clearly far from accurate. Hence, for loss-compensated metamaterials at zero temperature, we find a clear failure of the quantum optical effective-index theory to predict an accurate power spectrum for loss-compensated metamaterials. A new effective theory is needed that also accurately describes the amount of noise photons in metamaterials.

### 7.2. Power Spectra for Loss-Loss and Gain-Gain MM

**Figure 5:**
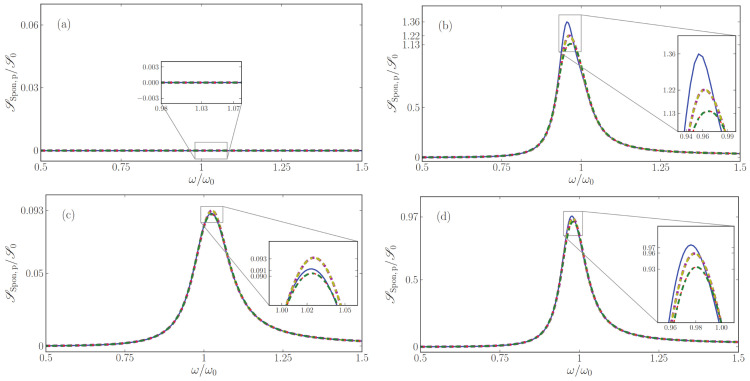
The spontaneous-emission power spectrum of the noise photons (A20), in units of $S_{0}=\hbar \omega_{0}^{3}/4\pi \varepsilon_{0}c^{3}$, for *p*-polarized light exiting the MM at 60 degrees away from the normal direction. In all four panels, the QOEM theory and the quantum optical effective-index theory are compared to the exact multilayer calculation. For the exact multilayer calculation (solid blue curves), the loss and gain layers are described by Lorentz models (Equation (40)) with the same parameters as in Figure 2. Left and right panels correspond to loss-loss and gain-gain metamaterials with the geometry of Figure 6. The loss-loss and the gain-gain multilayers are maintained at zero temperature in panels (**a**,**b**), and at the elevated positive temperature $T=0.6\hbar \omega_{0}/k_{B}$ in panel (**c**), and at the elevated negative temperature $\left|T\right|=0.6\hbar \omega_{0}/k_{B}$ in (**d**). For the effective-index theories, red dotted and green dash-dotted curves present the numerical parameters as obtained from the scattering method (A15a) and the dispersion method (A17), respectively. For QOEM theory, magenta dashed and yellow dash-dotted lines correspond to these same classical effective parameter retrieval methods (A15a) and (A17). These effective parameters so obtained are also used to compute $N_{\mathrm{eff}}$.

In Figure 5, we compare power spectra for *p*-polarized light exiting at an angle of 60 degrees away from the normal of the MM, computed with the exact multilayer theory, with QOEM theory, and with the quantum optical effective-index theory. The left panels are for loss-loss MMs, the right panels for gain-gain MMs, upper panels for zero temperature and lower panels for elevated temperature. In panel (a) for the loss-loss multilayer at zero temperature, quantum noise can be neglected; therefore, just like in classical optics, the power spectrum of output light vanishes identically and perfect agreement between all curves is observed. By contrast, for the gain-gain multilayer at zero temperature in panel Figure 5b, the population of the two-level medium is fully inverted and the effects of quantum noise in the output cannot be neglected. The power spectrum of output light appears as a peak near $\omega_{0}$, which is associated with the resonance frequency of the dielectric functions of each layer. Away from resonance, both effective theories agree well with the exact calculation. Near resonance, there are differences on the order of a few percent between the exact multilayer calculation and the two effective-medium theories. As seen in the zoomed inset in Figure 5b, near the resonance, the QOEM theory is more accurate than the QOEI theory.

In panels Figure 5c for loss-loss MMs at a pretty high temperature and Figure 5d for gain-gain MMs at a negative temperature, the exact and the two effective power spectra again agree quite well, with only on resonance a few percent difference. Sufficiently far from the resonance when absorption is small, the thermal noise becomes negligibly small and the power spectrum of output noise photons is approximately zero. For the gain-gain multilayer, the amplitude of the peak in panel (d) is smaller than the one in (b), since amplification within gain layers is reduced by saturation effects. We checked (but do not show it here) that these results do not depend much on the typical parameters used in Figure 5. The overall message of Figure 5 is that both the QOEM theory and the quantum optical effective-index theory are quite accurate in describing power spectra of *p*-polarized light of loss-loss and gain-gain metamaterials, with the two effective theories almost equally accurate. Therefore, one can use either $N_{\mathrm{eff}}$ or $N_{\mathrm{th}}$ as the noise photon distribution in Equation (39).

To summarize our findings from this section, we compared for the first time the power spectra of metamaterials based on exact theory and on QOEM and effective-index theory. For loss-compensated metamaterials we find that the effective-index theory is manifestly inadequate, both for *s*- and *p*-polarized light. By contrast, our QOEM theory in a consistent way predicts that the quantum noise contribution $\langle F_{\sigma }^{†}\left(k,\omega \right)F_{\sigma^{\prime }}\left(k^{\prime },\omega^{\prime }\right)\rangle$ to the power spectrum of a layered metamaterial is given by Equation (39), but with the thermal distribution $N_{\mathrm{th}}\left(\omega ,|T|\right)$ replaced by the effective distribution $N_{\mathrm{eff},\;\sigma }\left(k,\omega ,T\right)$ of Equation (35). In the absence of loss compensation, i.e., for loss-loss and gain-gain metamaterials, we found that for *s*-polarized light the QOEM theory exactly coincides with the QOEI theory. For *p*-polarization, there is no such exact agreement in the absence of loss compensation, but numerically the differences between both effective theories are so small that it is essentially a matter of choice which one to use. For loss-compensated metamaterials, QOEM theory is the only accurate effective-medium theory.

## 8. Second Test: Propagation of Squeezed States

For the power spectra emitted by a metamaterial as discussed in Section 7, the input states of light were vacuum states, which have a classical analogue (no light). By contrast, here we analyze how well the difference effective-medium theories describe the output quantum states of light when the input states have no classical analogues. This will serve as a useful independent test of the accuracy of the effective-medium theories. We will study the propagation of squeezed states of light through the metamaterial, generalizing Ref. [59] to arbitrary angles of incidence. The main question is how well quantum properties of the incoming state are preserved in the output, given that there is quantum noise in the metamaterial. We compare the answers to this question as obtained by exact multilayer theory and by quantum effective-index and effective medium theories. Most importantly, we investigate whether the QOEM theory that so accurately described power spectra in Section 7 also describes the propagation of squeezed states well.

**Figure 6:**
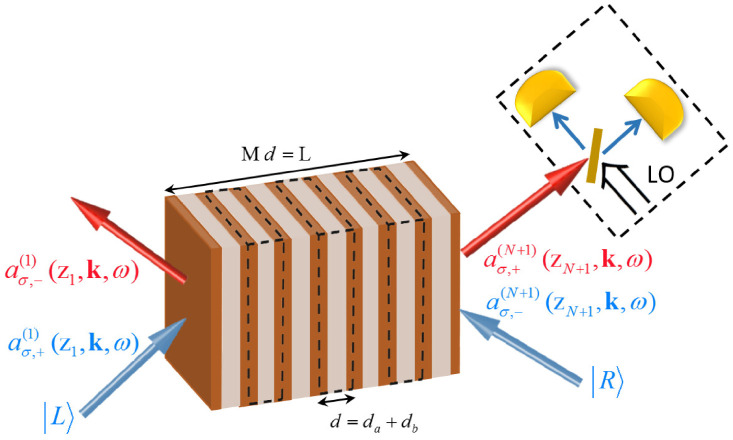
(Color online) Scheme of the loss-compensated multilayer medium in air. It has alternating layers with thicknesses $d_{a,b}$ that are arranged symmetrically. The two outermost layers have widths $d_{a}/2$, which makes the medium finite periodic with *M* unit cells. The amplifying and absorbing layers are described by the Lorentz model (Equation (40)), with parameters $\omega_{p_{a}}/\omega_{0}=0.3$, $\gamma_{a}/\omega_{0}=0.1$ for the lossy layers, and $\omega_{p_{b}}/\omega_{0}=0.25$, $\gamma_{b}/\omega_{0}=0.15$ for the layers with gain. We choose $d_{a,b}\omega_{0}/c=0.1$ and M=5. The incident squeezed vacuum state |L〉 has the squeeze strength $\zeta_{\sigma }=0.2$ and phase $\phi_{\sigma ,\zeta }=2\phi_{\sigma ,\mathrm{LO}}-\frac{5}{2}$, while the squeezed vacuum state |R〉 has the same strength $\xi_{\sigma }=0.2$ with $\phi_{\sigma ,\xi }=2\phi_{\sigma ,\mathrm{LO}}-2$, all assumed to be frequency independent. The outgoing light on the right-hand side of the multilayer metamaterial is measured with a balanced homodyne detector, shown within the dashed box, which is assumed to co-rotate with the exit angle.

We will consider the same metamaterial for which we calculated power spectra before, as detailed in Figure 6. Since the tangential component k is preserved under propagation through the multilayer and since there is air on both sides of the metamaterial, the output state of light will emerge from the loss-compensated multilayer at the same angle θ. Squeezing, specifically quadrature squeezing, occurs when the variance in the quantum fluctuations in one of the quadrature components of the electromagnetic field drop below the vacuum level. Squeezed states have no classical analogues and their nonclassicality can be quantified by their associated normally ordered variances of the field operators [87]. Squeezed light can be produced by transmitting the radiation field through a nonlinear medium with a second-order nonlinearity $\chi^{\left(2\right)}$. Mathematically, the squeezed incident quantum states of light can be written as $|L\rangle =S_{\sigma }|0\rangle$ and $|R\rangle =S_{\sigma }^{\prime }|0\rangle$, with squeeze operators belonging to a fixed in-plane wavevector k given by

(41a) $$\begin{array}{ccc} & & \;S_{\sigma }=\exp\left\{\int_{0}^{\Delta \omega }d\omega \;\left[\xi_{\sigma }^{*}\left(k,\omega \right)e^{-i\phi_{\sigma ,\xi }\left(k,\omega \right)}\;a_{\sigma ,\;+}^{(1)\;†}\left(k,\omega \right)\;a_{\sigma ,\;+}^{(1)\;†}\left(k,2\Omega -\omega \right)-h.c.\right]\right\}, \end{array}$$

(41b) $$\begin{array}{ccc} & & \;S_{\sigma }^{\prime }=\exp\left\{\int_{0}^{\Delta \omega }d\omega \;\left[\zeta_{\sigma }^{*}\left(k,\omega \right)e^{-i\phi_{\sigma ,\zeta }\left(k,\omega \right)}\;a_{\sigma ,\;-}^{(N+1)\;†}\left(k,\omega \right)\;a_{\sigma ,\;-}^{(N+1)\;†}\left(k,2\Omega -\omega \right)-h.c.\right]\right\}. \end{array}$$

Here, the $a_{\sigma ,\;+}^{\left(1\right)}\left(k,\omega \right)$ and $a_{\sigma ,\;-}^{\left(N+1\right)}\left(k,\omega \right)$ are the photonic annihilation operators of the incident fields with polarization σ and the transverse wave vector k on the left- and right-hand sides of the multilayer slabs, respectively. It can be seen that the squeeze operators (41) correlate pairs of fixed-frequency modes on both sides of the frequency Ω. The amount of squeezing is controlled by the squeeze parameters $\xi_{\sigma }\left(k,\omega \right)$ and $\zeta_{\sigma }\left(k,\omega \right)$, which depend on the frequency, polarization, and angle of incidence. We specify the detector to be a balanced homodyne detector. It is well-known that squeezing can be measured in such a setup, where the signal field and a strong local oscillator are superimposed on a beam splitter, see Ref. [88] and the sketch in Figure 6. The measured quantity is the difference in the photo currents of two detectors placed in the output arms of the beam splitter, as represented by the operator [87,89]

(42) $${\hat{O}}_{\sigma }=i\int_{t_{0}}^{t_{0}+T_{0}}dt\;\left\{a_{\sigma ,\;+}^{(N+1)\;†}a_{\sigma ,\;\mathrm{LO}}-a_{\sigma ,\;\mathrm{LO}}^{†}a_{\sigma ,\;+}^{\left(N+1\right)}\right\},$$

where on the right-hand side we suppressed the (k,t)-dependence of operators for readability. The detector is assumed to be polarization selective, and it is switched on from time $t_{0}$ to $t_{0}+T_{0}$. The $a_{\sigma ,\;\mathrm{LO}}\left(t\right)$ and $a_{\sigma ,\;\mathrm{LO}}^{†}$ are the creation and annihilation operators of the local-oscillator field with polarization σ. This local-oscillator field is assumed to be a single-mode coherent light beam represented by the complex amplitude $\alpha_{\sigma ,\;\mathrm{LO}}\left(t\right)$ that equals $F_{\mathrm{LO}}^{1/2}\exp\left[-i\left(\omega_{\mathrm{LO}}t-\phi_{\sigma ,\mathrm{LO}}\right)\right]$, in terms of a flux $F_{\mathrm{LO}}$, a phase $\phi_{\sigma ,\mathrm{LO}}$, and the frequency $\omega_{\mathrm{LO}}$. With the usual assumption that the local-oscillator field is much more intense than the signal field, the measurement operator ${\hat{O}}_{\sigma }$ of Equation (42) can be written as

(43) $${\hat{O}}_{\sigma }=F_{\mathrm{LO}}^{1/2}\int_{t_{0}}^{t_{0}+T_{0}}dt\;E_{\sigma }\left(\phi_{\sigma ,\mathrm{LO}},k,t\right),$$

where the operator $E_{\sigma }\left(\phi_{\mathrm{LO}},k,t\right)$ that equals $a_{\sigma ,\;+}^{\left(N+1\right)}\left(k,t\right)\exp\left[i\left(\omega_{\mathrm{LO}}t-\phi_{\sigma ,\mathrm{LO}}-\pi /2\right)\right]+h.c$ is one quadrature operator of the output field with wave vector k and polarization σ that exits the loss-compensated metamaterial on the right in Figure 6. Balanced homodyne detection allows to measure a single quadrature component of the scattered field [88]. From the above definitions, the variance in the difference photocount in a narrow-bandwidth homodyne detector can be obtained as [89,90]

(44) 〈[ΔEσ(ϕσ,LO,k,ωLO)]2〉out=1+2〈aσ,+(N+1)†(k,ωLO),aσ,+(N+1)(k,ωLO)〉+2Re[〈aσ,+(N+1)†(k,ωLO),aσ,+(N+1)†(k,ωLO)〉e2iϕσ,LO],

where the short-hand notation 〈C,D〉≡〈CD〉−〈C〉〈D〉 has been introduced for a correlation. The scattered output state is squeezed if its photocount variance is smaller than that of the vacuum state value [87]. The homodyne electric-field operator has a variance (44) equal to unity for the vacuum state. Therefore, the amount of squeezing is gauged by the difference between this variance and unity. We will now calculate the quadrature variances in Equation (44) in three ways: using the exact multilayer theory, the effective-index theory, and by the QOEM theory. In all three cases, we make use of the commutation relation Equations (13) and the definition of the squeezing parameters (41). We start calculating the variances, Equation (44), with the multilayer theory, where the crucial relation between input and output operators is given by (23). This will result in rather long expressions, which is one of the reasons to try to find simple but accurate effective theories also in quantum optics. The two types of correlations in the variance in Equation (44) are given by

(45a) $$\begin{array}{ccc} & & \;\langle a_{\sigma ,\;+}^{(N+1)\;†}\left(k,\omega \right),a_{\sigma^{\prime },\;+}^{\left(N+1\right)}\left(k^{\prime },\omega^{\prime }\right)\rangle =\langle F_{\sigma ,\;+}^{†}\left(k,\omega \right)\;F_{\sigma ,\;+}\left(k^{\prime },\omega^{\prime }\right)\rangle +\delta_{\sigma \sigma^{\prime }}\delta \left(k-k^{\prime }\right)\delta \left(\omega -\omega^{\prime }\right)\\ & & \times \left(|T_{\sigma ,22}{\left(k,\omega \right)|}^{2}\;\sinh^{2}\xi_{\sigma }\left(k,\omega \right)+{\left|T_{\sigma ,21}\left(k,\omega \right)\right|}^{2}\;\sinh^{2}\zeta_{\sigma }\left(k,\omega \right)\right), \end{array}$$

(45b) $$\begin{array}{ccc} & & \;\langle a_{\sigma ,\;+}^{(N+1)\;†}\left(k,\omega \right),a_{\sigma^{\prime },\;+}^{(N+1)\;†}\left(k^{\prime },\omega^{\prime }\right)\rangle =\frac{1}{2}\delta_{\sigma \sigma^{\prime }}\delta \left(k-k^{\prime }\right)\delta \left(\omega +\omega^{\prime }-2\Omega \right)\\ & & \times \left(T_{\sigma ,22}^{*\;2}\left(k,\omega \right)\;\sinh2\xi_{\sigma }\left(k,\omega \right)e^{-i\phi_{\sigma ,\xi }\left(k,\omega \right)}+T_{\sigma ,21}^{*\;2}\left(k,\omega \right)\;\sinh2\zeta_{\sigma }\left(\omega \right)e^{-i\phi_{\sigma ,\zeta }\left(k,\omega \right)}\right). \end{array}$$

The homodyne signal depends on the noise as described by the operator $F_{\sigma ,\;+}\left(k,\omega \right)$, which represents the outgoing rightward-propagating noise field produced inside the multilayer medium. More specifically, the noise dependence is described by the expectation value $\langle F_{\sigma ,\;+}^{†}\left(k,\omega \right)\;F_{\sigma ,\;+}\left(k^{\prime },\omega^{\prime }\right)\rangle$, which is the same noise-photon flux that we also came across in the power spectrum (38). Thus, the effect of the quantum noise on the squeezing properties of output light can be fully characterized by the emitted noise photons. The reason why only the first of the two expressions in Equation (45) depends on the quantum noise is that the quantum noise is assumed to be in a thermal state for which $\langle F_{\sigma ,\;+}^{†}\left(k,\omega \right)\;F_{\sigma ,\;+}^{†}\left(k^{\prime },\omega^{\prime }\right)\rangle$ vanishes.

We will compare predictions of the homodyne signal made with the exact multilayer theory and with the two effective theories. For the multilayer theory, we can insert into Equation (45) the classical multilayer matrix $T_{\sigma }\left(k,\omega \right)$ of Equation (25a) and the multilayer noise flux ${\langle F_{\sigma ,\;+}^{†}\left(k,\omega \right)\;F_{\sigma ,\;+}\left(k^{\prime },\omega^{\prime }\right)\rangle }_{\mathrm{exact}}$ of Equation (A19) again. In both effective theories, on the other hand, the exact matrix coefficients of the input-output matrix are to be replaced by the corresponding elements of the effective matrix $T_{\mathrm{eff}}$ of Equation (28). Furthermore, in the effective-index theory the noise photon flux is given by Equation (39), while in the QOEM theory it is given by Equation (33).

**Figure 7:**
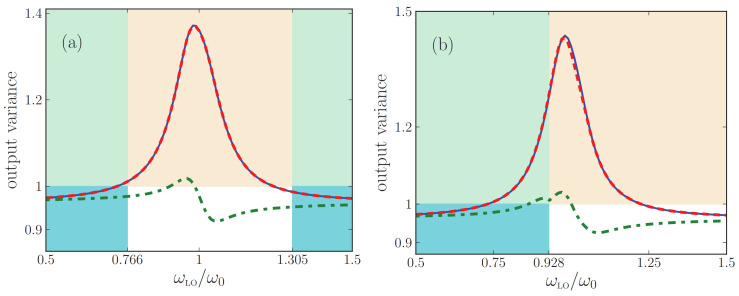
(Color online) For squeezed light incident at an angle of $\theta =30^{\circ }$ onto a loss-compensated multilayer metamaterial, a comparison of the predicted variances (44) as would be measured in balanced homodyne detection at a detection angle of also $30^{\circ }$. The metamaterial and the input states are described in Figure 6. Predictions with exact multilayer theory (blue solid line) are compared with the quantum optical effective-index theory (green dash-dotted) and quantum optical effective-medium theory (red dashed), for *s*-polarized input states of light in panel (**a**) and for *p*-polarization in (**b**).

**Figure 8:**
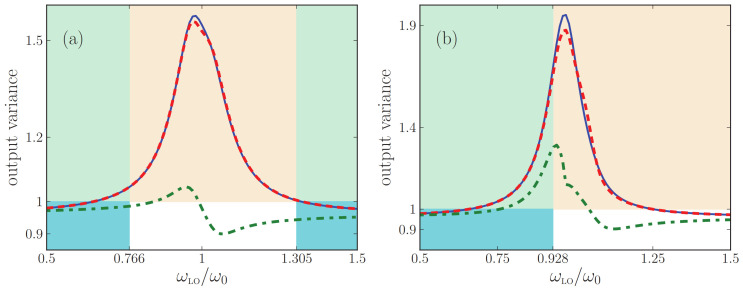
Same as Figure 7 but now for incident and detection angles of $\theta =60^{\circ }$.

In Figure 7, we compare the output squeezing spectrum predicted with the three theories, all for T=0, at an angle of 30 degrees away from the normal. In Figure 8, we show the same for an output angle of 60 degrees. For simplicity, we take the squeezing strengths $\xi_{\sigma }^{*}\left(k,\omega \right)$, $\zeta_{\sigma }^{*}\left(k,\omega \right)$ and phases $\phi_{\sigma ,\xi }$, $\phi_{\sigma ,\zeta }$ to be constant in the depicted frequency interval. We observe that the output squeezing spectrum is sensitive not only to the local-oscillator phase but also to the angle of incidence and the polarization. For this loss-compensated multilayer, the output squeezing spectrum shows a maximum exceeding unity in the vicinity of the resonance frequency. Noise photons destroy the squeezing property of the input field such that the output state will not at all be squeezed for most local-oscillator frequencies in the interval $\left[0.5\omega_{0},1.5\omega_{0}\right]$ shown in the figures. By contrast, in the same frequency interval, the quantum optical effective-index theory predicts the output light to be squeezed for almost all local-oscillator frequencies. In other words, the output state of light of the loss-compensated material is considerably noisier than that of the homogeneous slab with the same $\beta_{\sigma }$. Thus, Figure 7 and Figure 8 clearly illustrate the failure of the quantum optical effective-index theory for loss-compensated metamaterials. In Ref. [59], this failure was already established for normal incidence, and here we see that the agreement does not improve when detecting under an angle. The more important message from the figures is the very good agreement between the exact theory and QOEM effective theory that we generalized in this work, not only for normal incidence but now also under an angle, and both for *s*- and for *p*-polarized light. Small numerical differences between the exact theory and the QOEM theory occur only close to resonance and only for large incident angles.

The colors of the frequency intervals in Figure 7 and Figure 8 label net loss and net gain, exactly as before in Figure 3 and Figure 4. When loss is exactly compensated by gain, we saw in these earlier figures that $N_{\mathrm{eff},\;\sigma }\left(k,\omega ,T\right)$ diverges while the output intensity was continuous. Here, in Figure 7 and Figure 8, we see that, likewise, in homodyne detection the output variance is still continuous at those frequencies where $N_{\mathrm{eff},\;\sigma }\left(k,\omega ,T\right)$ diverges.

**Figure 9:**
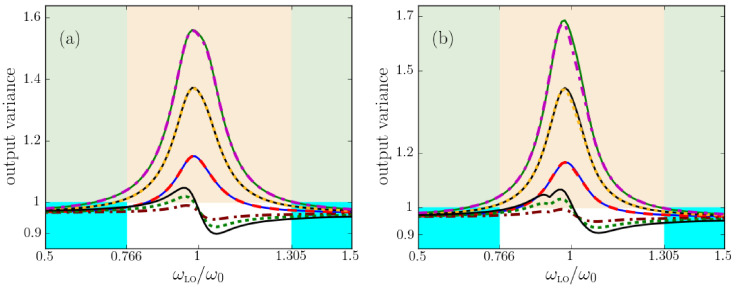
Same as Figure 7 but now for three different numbers of unit cells: M=3,6,9, in panel (**a**) for *s*-polarized and in (**b**) for *p*-polarized light. The three lowest curves describe QOEI theory, with the largest variation belonging to M=9. The three pairs of almost overlapping curves belong to M=3 (lower pair), M=6 (middle pair), and M=9 (upper pair). Each pair contains the exact multilayer theory (solid lines) with QOEM theory (dashed lines).

Finally, in Figure 9, we study how the number of unit cells affects the output variance. We see that for both polarizations, the output variance grows with the number of unit cells. In addition, the differences in the predicted output variances within the exact multilayer theory and QOEI theory grow as the number of layers is increased.

## 9. Discussion and Conclusions

We studied the propagation of quantum states of light through metamaterials, and showed that also in quantum optics an effective description of layered metamaterials can be given, for any angle of incidence and polarization. Quantum noise due to material loss or gain has an influence on the quantum states of light. We showed that for some metamaterials the effective index suffices to describe the quantum noise, while in other cases an additional effective-medium parameter is needed, namely, the effective noise-current density.

We tested our quantum optical effective-index theory (one effective parameter) and quantum optical effective-medium theory (two parameters) by calculating spectra and comparing with a full description of the multilayer metamaterial. For loss-compensated metamaterials, the gain regions emit noise photons, not described by the effective-index theory, that do affect the spectra. They have a similar effect on balanced homodyne detection measurements. We showed that our quantum optical effective-medium theory describes both the spectra and the homodyne signal well.

For normal incidence, we found earlier that the quantum noise of passive metamaterials can be described in terms of the effective index, and loss-compensated metamaterials require the additional parameter. We now found that this also holds exactly for *s*-polarized light at all angles of incidence, but for *p*-polarized light the additional parameter is also needed for passive systems. For all angles of incidence and polarizations, we derived expressions for the new effective parameter.

Our results can be readily generalized to magnetic layered metamaterials. For metamaterials not composed of multilayers, more work would be needed to derive the effective noise current density. Metasurfaces with gain will similarly require a description in quantum optics that describes the quantum noise associated with the gain. Another interesting open question is whether the current effective-medium theories suffice to describe higher-order measurements, for example bunching or anti-bunching in intensity correlation measurements, for quantum states of light that propagated through metamaterials.

## Data Availability

All data included in this study are available upon request by contact with the corresponding author.

## Appendix A. Green Function for Multilayers with Gain and Loss

Without repeating the derivation, we present the result of Ref. [76], namely, the classical Green function of a multilayer medium with both gain and loss. This is a generalization of the result by Tomaš for lossy dielectric multilayers [85], whose notation we will follow. We also address some subtleties arising only for media with gain.

The Green function $G(x,x^{\prime },\omega )$ was introduced in Section 2. For multilayer media, it is convenient to write it in the same mixed Fourier representation as is done for the electric-field operator in Section 3:

(A1) $$\begin{array}{c} G(x,x^{\prime },\omega )=\frac{1}{2\pi }\int d^{2}k\;e^{ik\cdot (\rho -\rho^{\prime })}G\left(k,z,z^{\prime },\omega \right). \end{array}$$

Here, the Green tensor $G(k,z,z^{\prime },\omega )$ assumes two different forms, depending on whether *z* and $z^{\prime }$ are located in the same layer or not. For $z^{\prime }$ in layer *j* it is given by [76,85]

(A2a) $$\begin{array}{ccc} G(k,z,z^{\prime },\omega ) & = & \frac{-1}{2\pi k_{j}^{2}}\delta \left(z-z^{\prime }\right)\hat{z}\hat{z}+\frac{i}{4\pi \beta_{j}}\sum_{\sigma =s}^{p}\xi_{\sigma }\frac{e^{i\beta_{j}d_{j}}}{D_{\sigma }^{j}}\\ & & \times [E_{\sigma \;>}^{\left(j\right)}\left(k,\omega ;z\right)E_{\sigma \;<}^{\left(j\right)}\left(-k,\omega ;z^{\prime }\right)\Theta \left(z-z^{\prime }\right)+E_{\sigma \;<}^{\left(j\right)}\left(k,\omega ;z\right)E_{\sigma \;>}^{\left(j\right)}\left(-k,\omega ;z^{\prime }\right)\Theta \left(z^{\prime }-z\right)],\;\;\;z\;\;\;\mathrm{also}\;\mathrm{in}\;\mathrm{layer}\;j \end{array}$$

(A2b) $$\begin{array}{ccc} G(k,z,z^{\prime },\omega ) & = & \frac{i}{4\pi \beta_{n}}\sum_{\sigma =s}^{p}\xi_{\sigma }\frac{t_{\sigma }^{n/j}e^{i(\beta_{j}d_{j}+\beta_{n}d_{n})}}{D_{\sigma }^{j}}\\ & & \times \left[\frac{E_{\sigma \;>}^{\left(n\right)}\left(k,\omega ;z\right)}{D_{\sigma ,+}^{n/j}}E_{\sigma \;<}^{\left(j\right)}\left(-k,\omega ;z^{\prime }\right)\Theta \left(n-j\right)+\frac{E_{\sigma \;<}^{\left(n\right)}\left(k,\omega ;z\right)}{D_{\sigma ,-}^{n/j}}E_{\sigma \;>}^{\left(j\right)}\left(-k,\omega ;z^{\prime }\right)\Theta \left(j-n\right)\right],\;\;z\;\;\mathrm{in}\;\mathrm{layer}\;n\neq j \end{array}$$

where $\xi_{s}=-1$, $\xi_{p}=1$, and Θ(z) is the usual unit step function, and

(A3a) $$\begin{array}{ccc} E_{\sigma \;>}^{\left(j\right)}\left(k,\omega ;z\right) & = & e_{\sigma ,+}^{\left(j\right)}\left(q\right)e^{i\beta_{j}\left(z-d_{j}\right)}+r_{\sigma ,+}^{j}e_{\sigma ,-}^{\left(j\right)}\left(k\right)e^{-i\beta_{j}\left(z-d_{j}\right)}, \end{array}$$

(A3b) $$\begin{array}{ccc} E_{\sigma \;<}^{\left(j\right)}\left(k,\omega ;z\right) & = & e_{\sigma ,-}^{\left(j\right)}\left(k\right)e^{-i\beta_{j}z}+r_{\sigma ,-}^{j}e_{\sigma ,+}^{\left(j\right)}\left(k\right)e^{i\beta_{j}z}. \end{array}$$

Here, σ stands for *s*- or *p*-polarization, and $e_{s,\pm }^{\left(j\right)}=\left(\hat{k}\times \hat{z}\right)$ and $e_{p,\pm }^{\left(j\right)}=\frac{-1}{k_{j}}\left(|k\right|\hat{z}\pm \beta_{j}\hat{k})$ are the polarization vectors for *s*- and *p*-polarized waves propagating in the positive-/negative-*z* direction, where $k_{j}\equiv \sqrt{\omega^{2}\varepsilon_{j}\left(\omega \right)/c^{2}}=k_{j}^{\prime }+ik_{j}^{\prime \prime }$ and

(A4) $$\beta_{j}\left(k,\omega \right)=\sqrt{\varepsilon_{j}\left(\omega \right)\omega^{2}/c^{2}-k^{2}}=\beta_{j}^{\prime }+i\beta_{j}^{\prime \prime }$$

is the normal component of the wave vector in layer *j*th and k the in-plane wave vector. Other quantities in Equations (A2) that still need to be defined are

(A5a) $$\begin{array}{ccc} D_{\sigma }^{j} & = & 1-r_{\sigma ,-}^{j}r_{\sigma ,+}^{j}e^{2i\beta_{j}d_{j}}, \end{array}$$

(A5b) $$\begin{array}{ccc} D_{\sigma ,\pm }^{n/j} & = & 1-r_{\sigma ,\pm }^{n}r_{\sigma }^{n\mp 1/j}e^{2i\beta_{n}d_{n}}, \end{array}$$

where $r_{\sigma ,-}^{j}$ and $r_{\sigma ,+}^{j}$ are the Fresnel coefficients for reflection at the left/right boundary of layer *j*. In addition, $t_{\sigma }^{n/j}$ and $r_{\sigma }^{n/j}$ are the transmission and reflection coefficients between the layers *n* and *j*.

The Green function (A2) is hereby defined, but not yet automatically well-defined: it is a known issue that the normal component of the wave vector Equation (A4) for active multilayer media is not automatically well defined even if the refractive index is well-defined: although the refractive index has no branch points in the upper half-plane because of causality, $\beta_{j}\left(k,\omega \right)$ may have branch points there [91]. If so, one can observe two types of absolute and convective instabilities [92,93], which, respectively, correspond to the exponential growth of the field amplitude in time at a certain spatial point and blowing up the electromagnetic energy with time along the propagation direction. Therefore, $\beta_{j}\left(k,\omega \right)$ looses its usual physical interpretation as the propagation constant in the normal direction. In this manner, electromagnetic waves can propagate an infinite distance perpendicularly to the *z*-axis, and, therefore, pick up an infinite amount of gain, before arriving at any other plane z=const. These instabilities could be eliminated by making use of an active medium with a very high threshold gain and also limiting the extent of the active medium in the transverse direction. Unlike Refs. [91,92,93], we here focus on the system dynamics in stable regimes and only consider active media without branch points where $\beta_{j}\left(\omega \right)$ is meaningful for real frequencies. In that case, the signs of $\beta_{j}^{\prime }$ and $\beta_{j}^{\prime \prime }$ are identical to those of Re[εj(ω)] and Im[εj(ω)], respectively (see Refs. [91,92,93]).

## Appendix B. Langevin Equations for Amplitude Operators

On the one hand, the electric-field operator is expressed in Equation (22) in terms of the amplitude operators. On the other hand, it can be expressed in terms of the Green function of the medium, analogous to Equation (19). By equating the two expressions and using the explicit expression for the Green function of a multilayer medium as given in Appendix A, it follows that the *z*-dependence of the amplitude operators $a_{\sigma ,\pm }^{\left(j\right)}\left(z,k,\omega \right)$ is governed by quantum Langevin equations

(A6) $$\begin{array}{c} \frac{\partial a_{\sigma ,\pm }^{\left(j\right)}\left(z,k,\omega \right)}{\partial z}=\mp \beta_{j}^{\prime \prime }\;a_{\sigma ,\pm }^{\left(j\right)}\left(z,k,\omega \right)\pm \frac{\sqrt{2|\beta_{j}^{\prime \prime }|}}{i}\;e^{\mp i\beta_{j}^{\prime }z}\;f_{\sigma ,\pm }^{\left(j\right)}\left(z,k,\omega \right), \end{array}$$

so that the operators $a_{\sigma ,\pm }^{\left(j\right)}\left(z,k,\omega \right)$ and $a_{\sigma ,\pm }^{\left(j\right)}\left(z^{\prime },k,\omega \right)$ for different space points within the same *j*th layer are related as

(A7) $$\begin{array}{c} a_{\sigma ,\pm }^{\left(j\right)}\left(z,k,\omega \right)=e^{\mp \beta_{j}^{\prime \prime }\left(z-z^{\prime }\right)}a_{\sigma ,\pm }^{\left(j\right)}\left(z^{\prime },k,\omega \right)\;\pm \frac{\sqrt{2|\beta_{j}^{\prime \prime }|}}{i}\;e^{\mp \beta_{j}^{\prime \prime }z}\int_{z^{\prime }}^{z}dz^{\prime \prime }\;\;e^{\mp i\beta_{j}^{\prime }z^{\prime \prime }}\;f_{\sigma ,\pm }^{\left(j\right)}\left(z^{\prime \prime },k,\omega \right). \end{array}$$

Here, the $f_{\sigma ,\pm }^{\left(j\right)}\left(\pm z^{\prime },k,\omega \right)=f^{\left(j\right)}\left(\pm z^{\prime },k,\omega \right)\cdot e_{\sigma ,\pm }^{\left(j\right)}\left(k\right)$ are bosonic field operators that play the role of fundamental variables of the electromagnetic field and medium, and $f^{\left(j\right)}\left(\pm z^{\prime },k,\omega \right)$ is the partial Fourier transform of f(x,ω) in layer *j* of Equation (19). They satisfy the commutation relations

(A8a) $$\begin{array}{c} \;\left[f_{\sigma ,\pm }^{\left(j\right)}\left(z,k,\omega \right),f_{\sigma^{\prime },\pm }^{(j^{\prime })\;†}\left(z^{\prime },k^{\prime },\omega^{\prime }\right)\right]=\varrho_{\sigma ,+}^{\left(j\right)}\mathrm{sgn}[\varepsilon_{j,\;I}\left(\omega \right)]\delta_{jj^{\prime }}\delta_{\sigma \sigma^{\prime }}\delta \left(z-z^{\prime }\right)\delta \left(\omega -\omega^{\prime }\right)\delta \left(k-k^{\prime }\right), \end{array}$$

(A8b) $$\begin{array}{c} \;\left[f_{\sigma ,\pm }^{\left(j\right)}\left(z,k,\omega \right),f_{\sigma^{\prime },\mp }^{(j^{\prime })\;†}\left(z^{\prime },k^{\prime },\omega^{\prime }\right)\right]=\varrho_{\sigma ,-}^{\left(j\right)}\mathrm{sgn}[\varepsilon_{j,\;I}\left(\omega \right)]\delta_{jj^{\prime }}\delta_{\sigma \sigma^{\prime }}\delta \left(z-z^{\prime }\right)\delta \left(\omega -\omega^{\prime }\right)\delta \left(k-k^{\prime }\right). \end{array}$$

Here, the coefficient $\rho_{\sigma ,\pm }^{\left(j\right)}$ is defined to be equal to unity for *s*-polarization (i.e., for σ=s), while it is equal to $(k^{2}\pm |\beta_{j}{|}^{2})/|k_{j}{|}^{2}$ for σ=p. Notice that the plus and minus subscripts in this coefficient $\rho_{\sigma ,\pm }^{\left(j\right)}$ do not correspond to a propagation direction, but rather to two identical (+) and opposite (−) propagation directions.

*Special Cases: Incoming Fields.—*The amplitude operators of the incoming fields $a_{\sigma ,+}^{\left(1\right)}\left(z,k,\omega \right)$ and $a_{\sigma ,-}^{\left(N+1\right)}\left(z,k,\omega \right)$ in Equation (23) are defined within the space intervals $-\infty <z\leq z_{1}$ and $z_{N+1}\leq z<\infty$, respectively, see the sketch in Figure 1. The explicit forms of these input operators can be obtained with the use of Equation (A7) as

(A9a) $$\begin{array}{ccc} a_{\sigma ,+}^{\left(1\right)}\left(z,k,\omega \right) & = & \frac{1}{i}\sqrt{2|\beta_{1}^{\prime \prime }|}\;\;e^{-\beta_{1}^{\prime \prime }z}\int_{-\infty }^{z}dz^{\prime }\;e^{-i\beta_{1}z^{\prime }}\;f_{\sigma ,+}^{\left(1\right)}\left(z^{\prime },k,\omega \right), \end{array}$$

(A9b) $$\begin{array}{ccc} a_{\sigma ,-}^{\left(N+1\right)}\left(z,k,\omega \right) & = & \frac{1}{i}\sqrt{2|\beta_{N+1}^{\prime \prime }|}\;\;e^{\beta_{N+1}^{\prime \prime }z}\int_{z}^{\infty }dz^{\prime }\;e^{i\beta_{N+1}z^{\prime }}\;f_{\sigma ,-}^{\left(N+1\right)}\left(z^{\prime },k,\omega \right). \end{array}$$

## Appendix C. Three-Step Procedure to Derive Input-Output Operator Relations

*Step 1.—*The first step is readily found by realizing that Equation (A7) for $z=z_{j}$ can be written in matrix form as

(A10) $$\begin{array}{c} \left(\begin{array}{c} a_{\sigma ,+}^{\left(j\right)}\left(z_{j},k,\omega \right)\\ a_{\sigma ,-}^{\left(j\right)}\left(z_{j},k,\omega \right) \end{array}\right)=R_{\sigma }^{\left(j\;\right)}\left(\begin{array}{c} a_{\sigma ,+}^{\left(j\right)}\left(z_{j-1},k,\omega \right)\\ a_{\sigma ,-}^{\left(j\right)}\left(z_{j-1},k,\omega \right) \end{array}\right)+\left(\begin{array}{c} c_{\sigma ,+}^{\left(j\right)}\left(k,\omega \right)\\ c_{\sigma ,-}^{\left(j\right)}\left(k,\omega \right) \end{array}\right), \end{array}$$

where $R_{\sigma }^{\left(j\;\right)}$ is a diagonal 2×2 matrix with $R_{\sigma ,11}^{\left(j\;\right)}=1/R_{\sigma ,22}^{\left(j\;\right)}=e^{-\beta_{j}^{\prime \prime }d_{j}}$. The quantum noise operators in this matrix Equation (A10) are given by

(A11) $$\begin{array}{c} c_{\sigma ,\pm }^{\left(j\right)}\left(k,\omega \right)=\pm \frac{\sqrt{2|\beta_{j}^{\prime \prime }|}}{i}\;e^{\mp \beta_{j}^{\prime \prime }z_{j}}\int_{z_{j-1}}^{z_{j}}dz^{\prime }\;\;e^{\mp i\beta_{j}^{\prime }z^{\prime }}\;f_{\sigma ,\pm }^{\left(j\right)}\left(z^{\prime },k,\omega \right), \end{array}$$

and, evidently, these inhomogeneous terms in the matrix relation (A10) are the qualitative novelty as compared to the standard transfer-matrix analysis of multilayer media in classical electrodynamics. Recalling the commutation relations (A8), the operators $c_{\sigma ,\pm }^{\left(j\right)}\left(k,\omega \right)$ are found to satisfy the commutation relations

(A12a) $$\begin{array}{ccc} \left[c_{\sigma ,\pm }^{\left(j\right)}\left(k,\omega \right),c_{\sigma^{\prime },\pm }^{(j)\;†}\left(k^{\prime },\omega^{\prime }\right)\right] & = & \;2\;\rho_{\sigma ,+}^{\left(j\right)}\;e^{\mp \beta_{j}^{\prime \prime }d_{j}}\sinh\left(\beta_{j}^{\prime \prime }d_{j}\right)\;\delta_{\sigma \sigma^{\prime }}\\ & & \times \delta \left(\omega -\omega^{\prime }\right)\delta^{2}\left(k-k^{\prime }\right), \end{array}$$

(A12b) $$\begin{array}{ccc} \left[c_{\sigma ,\pm }^{\left(j\right)}\left(k,\omega \right),c_{\sigma^{\prime },\mp }^{(j)\;†}\left(k^{\prime },\omega^{\prime }\right)\right] & = & \;-\frac{2\beta_{j}^{\prime \prime }}{\beta_{j}^{\prime }}\;\rho_{\sigma ,-}^{\left(j\right)}\;e^{\mp i\beta_{j}^{\prime }\left(z_{j}+z_{j-1}\right)}\\ & & \times \sin\left(\beta_{j}^{\prime }d_{j}\right)\;\delta_{\sigma \sigma^{\prime }}\delta \left(\omega -\omega^{\prime }\right)\delta^{2}\left(k-k^{\prime }\right). \end{array}$$

*Step 2.—*In the second step, we relate the operators $a_{\sigma ,\pm }^{\left(j+1\right)}\left(z_{j},k,\omega \right)$ and $a_{\sigma ,\pm }^{\left(j\right)}\left(z_{j},k,\omega \right)$ in neighboring layers across the interface at $z_{j}$ to each other using the form Equation (A2) for the Green function $G(k,z,z^{\prime },\omega )$ for positions $z,z^{\prime }$ in neighboring layers. This Green function already, by construction, respects the Maxwell boundary conditions that the tangential components of the electric and magnetic fields be continuous. We obtain the operator matrix relation

(A13) $$\left(\begin{array}{c} a_{\sigma ,+}^{\left(j+1\right)}\left(z_{j},k,\omega \right)\\ a_{\sigma ,-}^{\left(j+1\right)}\left(z_{j},k,\omega \right) \end{array}\right)=S_{q}^{\left(j\right)}\left(\begin{array}{c} a_{\sigma ,+}^{\left(j\right)}\left(z_{j},k,\omega \right)\\ a_{\sigma ,-}^{\left(j\right)}\left(z_{j},k,\omega \right) \end{array}\right),$$

which also holds for classical amplitudes and where the matrix $S_{\sigma }^{\left(j\right)}$ is given by

(A14) $$S_{\sigma }^{\left(j\right)}=\frac{1}{2\beta_{j}}\sqrt{\frac{\beta_{j}^{\prime }}{\beta_{j+1}^{\prime }}}\left(\begin{array}{c} \left(\beta_{j+1}\kappa_{\sigma ,j/j+1}+\beta_{j}\kappa_{\sigma ,j+1/j}\right)e^{i\left(\beta_{j}^{\prime }-\beta_{j+1}^{\prime }\right)z_{j}}\;\;\;\;\;\;\;\left(\beta_{j+1}\kappa_{\sigma ,j/j+1}-\beta_{j}\kappa_{\sigma ,j+1/j}\right)e^{-i\left(\beta_{j}^{\prime }+\beta_{j+1}^{\prime }\right)z_{j}}\\ \left(\beta_{j+1}\kappa_{\sigma ,j/j+1}-\beta_{j}\kappa_{\sigma ,j+1/j}\right)e^{i\left(\beta_{j}^{\prime }+\beta_{j+1}^{\prime }\right)z_{j}}\;\;\;\;\;\;\;\left(\beta_{j+1}\kappa_{\sigma ,j/j+1}+\beta_{j}\kappa_{\sigma ,j+1/j}\right)e^{-i\left(\beta_{j}^{\prime }-\beta_{j+1}^{\prime }\right)z_{j}} \end{array}\right),$$

in which $\kappa_{s,j/j+1}=1$ and $\kappa_{p,j/j+1}=k_{j}/k_{j+1}$.

*Step 3.—*In the third, final step, we invoke Equations (A10) and (A13) alternatingly and repeatedly, until we finally obtain the operator of the outgoing fields to the leftmost and rightmost layers, respectively, $a_{\sigma ,-}^{\left(1\right)}\left(z_{1},k,\omega \right)$ and $a_{\sigma ,+}^{\left(N+1\right)}\left(z_{N},k,\omega \right)$, in terms of the two incoming fields $a_{\sigma ,+}^{\left(1\right)}\left(z_{1},k,\omega \right)$ and $a_{\sigma ,-}^{\left(N+1\right)}\left(z_{N},k,\omega \right)$, as well as the noise fields. The sought input–output relation for the amplitude operators is thereby obtained as Equation (23) of the main text.

## Appendix D. Methods to Obtain Classical Effective Parameters

*Scattering method.—*The scattering method developed by Smith and coworkers [11,12,18] has proved extremely useful. The idea is to fit the scattering properties of a metamaterial by those of a homogeneous medium, with values for the effective parameters that give the best fit. Finding equivalent bulk parameters in this way is solving an inverse problem. This approach has been generalized to oblique incidence [16] by assuming that the effective medium can be fully characterized by $\beta_{\mathrm{eff}}$, the wave-vector component in normal direction (here: $\hat{z}$-direction). In fact, for oblique incidence, there is no need to introduce the effective refractive index because all details of wave propagation follow from this parameter $\beta_{\mathrm{eff}}$. What is more, the refractive index may lose its physical meaning and may even become discontinuous, due to the branch cut of the complex square root [16,94]. By retrieving the normal wave vector component $\beta_{\mathrm{eff}}$ and the generalized impedance $Z_{\mathrm{eff},\;\sigma }=\beta_{\mathrm{eff},\;\sigma }/\epsilon_{\sigma }$ from the reflection and transmission coefficients (29a) and (29b) of a homogenous medium with thickness L, the effective wave parameters for both polarizations *s* and *p* are derived as

(A15a) $$\begin{array}{ccc} & & \beta_{\mathrm{eff},\;\sigma }\;L=\pm \arccos\left(\frac{1-r_{\mathrm{eff},\;\sigma }^{2}+t_{\mathrm{eff},\;\sigma }^{{}^{\prime }2}}{2t_{\mathrm{eff},\;\sigma }^{\prime }}\right), \end{array}$$

(A15b) $$\begin{array}{ccc} & & Z_{\mathrm{eff},\;\sigma }=\pm \beta_{0}\;\sqrt{\frac{{\left(r_{\mathrm{eff},\;\sigma }-1\right)}^{2}-t_{\mathrm{eff},\;\sigma }^{{}^{\prime }2}}{{\left(r_{\mathrm{eff},\;\sigma }+1\right)}^{2}-t_{\mathrm{eff},\;\sigma }^{{}^{\prime }2}}}, \end{array}$$

where $t_{\mathrm{eff},\;\sigma }^{\prime }=T_{\sigma ,21}\exp\left(i\beta_{0}L\right)$ is the modified transmission amplitude and the signs ± are chosen independently based on physical considerations [16]. In general, the multiple branches associated with the inverse cosine of Equation (A15a) make the unambiguous determination of the normal wave vector component $\beta_{\mathrm{eff},\;\sigma }$ difficult [18]. However, the ambiguity will not arise in our calculations since, for simplicity, we only consider situations where the wavelength within the medium is much larger than the multilayer length *L*.

*Dispersion method.—*Alternatively one can identify values for effective parameters using the dispersion method: the effective parameters of a periodic bi-layer system with permittivity functions $\varepsilon_{a}\left(\omega \right)$, $\varepsilon_{b}\left(\omega \right)$ and thicknesses $d_{a,b}$ are obtained from the Bloch dispersion relation

(A16) $$\begin{array}{c} \cos\left(\beta_{\mathrm{eff},\;\sigma }d\right)=\cos\left(\beta_{a}d_{a}\right)\cos\left(\beta_{b}d_{b}\right)-\frac{1}{2}\left(\frac{\beta_{a,\sigma }}{\beta_{b,\sigma }}+\frac{\beta_{b,\sigma }}{\beta_{a,\sigma }}\right)\sin\left(\beta_{a}d_{a}\right)\sin\left(\beta_{b}d_{b}\right) \end{array}$$

in the long-wavelength limit. We describe *s*- and *p*-polarized light at the same time, since $\beta_{j,\sigma }$ stands for $\beta_{j,s}=\beta_{j}$ and $\beta_{j,p}$ for $\beta_{j}/\varepsilon_{j}$, while *d* equals the total thickness $d_{a}+d_{b}$ of the two bi-layers. By taking the Taylor expansion near the point (ω,k)=(0,0), we obtain the dispersion relation

(A17a) $$\begin{array}{ccc} & & \beta_{\mathrm{eff},\;s}^{2}+k^{2}=\varepsilon_{\mathrm{eff},\;⊥}\;\frac{\omega^{2}}{c^{2}}, \end{array}$$

(A17b) $$\begin{array}{ccc} & & \frac{\beta_{\mathrm{eff},\;p}^{2}}{\varepsilon_{\mathrm{eff},\;⊥}}+\frac{k^{2}}{\varepsilon_{\mathrm{eff},\;∥}}=\frac{\omega^{2}}{c^{2}}, \end{array}$$

in terms of two important effective parameters, namely, $\varepsilon_{\mathrm{eff},\;⊥}=\left(\varepsilon_{a}d_{a}+\varepsilon_{b}d_{b}\right)/d$ and $\varepsilon_{\mathrm{eff},\;\|}=\left(\varepsilon_{a}\varepsilon_{b}d\right)/\left(\varepsilon_{b}d_{a}+\varepsilon_{a}d_{b}\right)$. The subscripts ⊥ and ‖ denote the directions perpendicular and parallel to the optical axis, which is here the *z*-axis, i.e., the optical axis points along the surface normal. Thus, the multilayer metamaterial effectively behaves as a uniaxial anisotropic medium [95] with the effective permittivity tensor $\bar{\bar{\varepsilon }}=diag\left(\varepsilon_{\mathrm{eff},\;⊥},\varepsilon_{\mathrm{eff},\;⊥},\varepsilon_{\mathrm{eff},\;\|}\right)$, where $\varepsilon_{\mathrm{eff},\;⊥}$($\varepsilon_{\mathrm{eff},\;\|}$) refers to the ordinary (extraordinary) permittivity of the effective medium. Since the in-plane wavevector *k* is conserved outside and inside the effective medium, it yields $k^{2}=\omega^{2}\sin^{2}\theta /c^{2}=\omega^{2}\varepsilon_{\mathrm{eff},\;s}\sin^{2}\theta_{\mathrm{eff}}/c^{2}$ and $\beta_{\mathrm{eff},\;s}^{2}=\omega^{2}\varepsilon_{\mathrm{eff},\;s}\cos^{2}\theta_{\mathrm{eff}}/c^{2}$ for *s*-polarization, where $\theta_{\mathrm{eff}}$ is the refracted angle inside the effective medium. Similar identities can be obtained for *p*-polarized light. Bearing these in mind, one can use Equation (A17) to write the effective dielectric functions for *s*- and *p*-polarized light in terms of the perpendicular and parallel effective permittivity functions. This gives $\varepsilon_{\mathrm{eff},\;s}=\varepsilon_{\mathrm{eff},\;⊥}$ for ordinary waves, which is angle-independent, and $\varepsilon_{\mathrm{eff},\;p}^{-1}\left(\theta_{\mathrm{eff}}\right)=\cos^{2}\theta_{\mathrm{eff}}/\varepsilon_{\mathrm{eff},\;⊥}+\sin^{2}\theta_{\mathrm{eff}}/\varepsilon_{\mathrm{eff},\;\|}$ for extraordinary waves. The latter exhibit a strong angle dependence varying from $\varepsilon_{\mathrm{eff},\;⊥}$ to $\varepsilon_{\mathrm{eff},\;\|}$ as $\theta_{\mathrm{eff}}$ is varied from $0^{\circ }$ to $90^{\circ }$. Remarkably, $\varepsilon_{\mathrm{eff},\;p}$ can also be written in terms of the incident angle θ as $\varepsilon_{\mathrm{eff},\;p}\left(\theta \right)=\varepsilon_{\mathrm{eff},\;⊥}+\sin^{2}\theta \left(1-\varepsilon_{\mathrm{eff},\;⊥}/\varepsilon_{\mathrm{eff},\;\|}\right)$.

## Appendix E. Quantum Noise Operators in QOEI Theory

The input–output relations (27) of QOEI theory feature two quantum noise terms $F_{\mathrm{eff},\;\sigma \pm }\left(k,\omega \right)$. These represent quantum noise associated with loss and gain and combinations thereof inside this effective medium. The right- (+) and left-going (−) components are given by

$$\begin{array}{ccc} F_{\mathrm{eff},\;\sigma -}\left(k,\omega \right) & = & \frac{-2i\beta_{0}\sqrt{2\beta_{\mathrm{eff},\;\sigma }^{\prime }\beta_{\mathrm{eff},\;\sigma }^{\prime \prime }\epsilon_{\sigma }/\beta_{0}^{\prime }}}{{\left(\beta_{\mathrm{eff},\;\sigma }+\epsilon_{\sigma }\beta_{0}\right)}^{2}-{\left(\beta_{\mathrm{eff},\;\sigma }-\epsilon_{\sigma }\beta_{0}\right)}^{2}\exp\left[2i\beta_{\mathrm{eff},\;\sigma }L\right]}\left(\left(\beta_{\mathrm{eff},\;\sigma }+\epsilon_{\sigma }\beta_{0}\right)\int_{0}^{L}dz^{\prime }e^{-i\beta_{\mathrm{eff},\;\sigma }z^{\prime }}f_{\mathrm{eff}\;\sigma ,+}\left(z^{\prime },k,\omega \right)\right. \end{array}$$

(A18a) $$\begin{array}{ccc} & & +\left.\left(\beta_{\mathrm{eff},\;\sigma }-\epsilon_{\sigma }\beta_{0}\right)\int_{0}^{L}dz^{\prime }e^{i\beta_{\mathrm{eff},\;\sigma }z^{\prime }}f_{\mathrm{eff}\;\sigma ,-}\left(z^{\prime },k,\omega \right)\right),\\ F_{\mathrm{eff},\;\sigma +}\left(k,\omega \right) & = & \frac{-2i\beta_{0}\sqrt{2\beta_{\mathrm{eff},\;\sigma }^{\prime }\beta_{\mathrm{eff},\;\sigma }^{\prime \prime }\epsilon_{\sigma }/\beta_{0}^{\prime }}\exp\left[i\left(\beta_{0}-\beta_{\mathrm{eff},\;\sigma }\right)L\right]}{{\left(\beta_{\mathrm{eff},\;\sigma }+\epsilon_{\sigma }\beta_{0}\right)}^{2}-{\left(\beta_{\mathrm{eff},\;\sigma }-\epsilon_{\sigma }\beta_{0}\right)}^{2}\exp\left[2i\beta_{\mathrm{eff},\;\sigma }L\right]}\left(\left(\beta_{\mathrm{eff},\;\sigma }+\epsilon_{\sigma }\beta_{0}\right)\int_{0}^{L}dz^{\prime }e^{i\beta_{\mathrm{eff},\;\sigma }z^{\prime }}f_{\mathrm{eff}\;\sigma ,-}\left(z^{\prime },k,\omega \right)\right. \end{array}$$

(A18b) $$\begin{array}{ccc} & & +\left.\left(\beta_{\mathrm{eff},\;\sigma }-\epsilon_{\sigma }\beta_{0}\right)e^{2i\beta_{\mathrm{eff},\;\sigma }L}\int_{0}^{L}dz^{\prime }e^{-i\beta_{\mathrm{eff},\;\sigma }z^{\prime }}f_{\mathrm{eff}\;\sigma ,+}\left(z^{\prime },k,\omega \right)\right), \end{array}$$

in terms of the fundamental bosonic operators *f* that were introduced in Section 2 and which, in an effectively homogeneous layer, satisfy the commutation relations (A8) in which ε is to be replaced by the effective permittivities $\varepsilon_{\mathrm{eff},\;\sigma }$ that are given in the previous Appendix.

## Appendix F. Flux of Noise Photons at T ≠ 0

By combining the expression (38) for the noise power spectrum with the input–output relations of the exact multilayer theory, in particular Equations (A11) and (24), we obtain the following exact expression for the flux of noise photons emitted from the loss-compensated multilayer

(A19) $$\begin{array}{ccc} {\langle F_{\sigma ,+}^{†}\left(k,\omega \right)\;\;F_{\sigma^{\prime },+}\left(k^{\prime },\omega^{\prime }\right)\rangle }_{\mathrm{exact}} & = & 2\sum_{j=2}^{N}\{\rho_{\sigma ,+}^{\left(j\right)}\;\sinh\left(\beta_{j}^{\prime \prime }d_{j}\right)\mathrm{sgn}\left[\varepsilon_{I,\;j}\left(\omega \right)\right]\left(|D_{\sigma \;21}^{\left(j\right)}{|}^{2}e^{-\beta_{j}^{\prime \prime }d_{j}}+{\left|D_{\sigma \;22}^{\left(j\right)}\right|}^{2}e^{\beta_{j}^{\prime \prime }d_{j}}\right)\\ & & \left.-\frac{|\beta_{j}^{\prime \prime }|}{\beta_{j}^{\prime }}\;\rho_{\sigma ,-}^{\left(j\right)}\;\sin\left(\beta_{j}^{\prime }d_{j}\right)\left(D_{\sigma \;21}^{(j)\;*}D_{\sigma \;22}^{\left(j\right)}e^{i\beta_{j}^{\prime }\left(z_{j}+z_{j-1}\right)}+D_{\sigma \;21}^{\left(j\right)}D_{\sigma \;22}^{(j)\;*}e^{-i\beta_{j}^{\prime }\left(z_{j}+z_{j-1}\right)}\right)\right\}\\ & & \times \left(N_{\mathrm{th}}\left(\omega ,T\right)\Theta \left[\varepsilon_{j,\;I}\left(\omega \right)\right]+\left(N_{\mathrm{th}}\left(\omega ,|T|\right)+1\right)\Theta \left[-\varepsilon_{j,\;I}\left(\omega \right)\right]\right)\delta_{\sigma \sigma^{\prime }}\delta \left(\omega -\omega^{\prime }\right)\delta \left(k-k^{\prime }\right). \end{array}$$

This formula is valid for all temperatures, and is used to produce Figure 3, Figure 4, Figure 5, Figure 7 and Figure 8. For loss-compensated metamaterials at zero temperature, the angle-dependent power spectrum is given by

(A20) $$\begin{array}{ccc} S_{\mathrm{Spon},\sigma }\left(\theta ,\omega \right) & = & \frac{-\hbar \omega^{3}\sin\theta }{2\pi \varepsilon_{0}c^{3}\;}\sum_{j=1}^{\frac{N}{2}-1}\{\varrho_{\sigma ,+}^{\left(2j+1\right)}\;\sinh\left(\beta_{2j+1}^{\prime \prime }d_{g}\right)\left(|D_{\sigma \;21}^{\left(2j+1\right)}{|}^{2}e^{-\beta_{2j+1}^{\prime \prime }d_{g}}+{\left|D_{\sigma \;22}^{\left(2j+1\right)}\right|}^{2}e^{\beta_{2j+1}^{\prime \prime }d_{g}}\right)\\ & & \;+\varrho_{\sigma ,-}^{\left(2j+1\right)}\;\frac{|\beta_{2j+1}^{\prime \prime }|}{\beta_{2j+1}^{\prime }}\;\sin\left(\beta_{2j+1}^{\prime }d_{g}\right)\left(D_{\sigma \;21}^{(2j+1)\;*}D_{\sigma \;22}^{\left(2j+1\right)}e^{i\beta_{2j+1}^{\prime }\left(z_{2j+1}+z_{2j}\right)}\right.\\ & & \;+\left.D_{\sigma \;21}^{\left(2j+1\right)}D_{\sigma \;22}^{(2j+1)\;*}e^{-i\beta_{2j+1}^{\prime }\left(z_{2j+1}+z_{2j}\right)}\right)\}. \end{array}$$
